# Constructing covalent organic nanoarchitectures molecule by molecule via scanning probe manipulation

**DOI:** 10.1038/s41557-021-00773-4

**Published:** 2021-09-02

**Authors:** Qigang Zhong, Alexander Ihle, Sebastian Ahles, Hermann A. Wegner, Andre Schirmeisen, Daniel Ebeling

**Affiliations:** 1 Institute of Applied Physics, Justus Liebig University Giessen, Giessen, Germany; 2 Center for Materials Research, Justus Liebig University Giessen, Giessen, Germany; 3 Institute of Organic Chemistry, Justus Liebig University Giessen, Giessen, Germany

**Keywords:** Chemistry, Nanoscience and technology

## Abstract

Constructing low-dimensional covalent assemblies with tailored size and connectivity is challenging yet often key for applications in molecular electronics where optical and electronic properties of the quantum materials are highly structure dependent. We present a versatile approach for building such structures block by block on bilayer sodium chloride (NaCl) films on Cu(111) with the tip of an atomic force microscope, while tracking the structural changes with single-bond resolution. Covalent homo-dimers in *cis* and *trans* configurations and homo-/hetero-trimers were selectively synthesized by a sequence of dehalogenation, translational manipulation and intermolecular coupling of halogenated precursors. Further demonstrations of structural build-up include complex bonding motifs, like carbon–iodine–carbon bonds and fused carbon pentagons. This work paves the way for synthesizing elusive covalent nanoarchitectures, studying structural modifications and revealing pathways of intermolecular reactions.

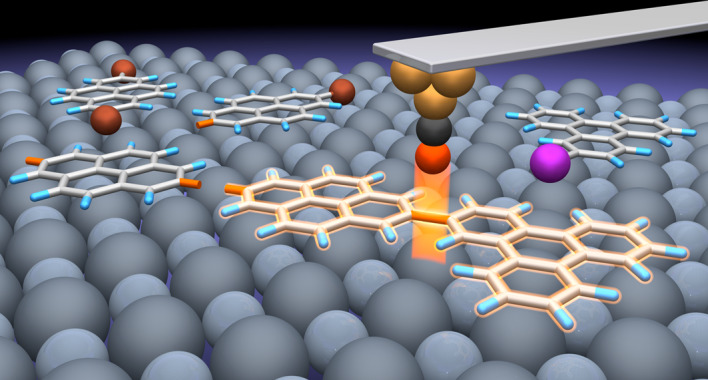

## Abstract (web summary)

Tailoring the size and connectivity of organic nanostructures is challenging but is often key in molecular electronics for tuning the properties of the quantum materials. Now an approach has been developed for building low-dimensional covalent architectures block by block on a surface by highly selective tip-induced intermolecular reactions.

## Main

The vision of assembling nanoarchitectures by controlled mechanical manipulation on an atom-by-atom or molecule-by-molecule basis has been a dream since its creation by R. Feynman and eventually led to the field of nanotechnology. Inspired by this, K. E. Drexler proposed the conceptual idea of molecular machines that would be capable of positioning reactants with atomic precision in order to guide chemical reactions, an idea that was heavily debated regarding its realizability^1^. The main counter argument presented is that the ‘fingers’ needed for orienting the atoms or molecules would be too ‘fat’ and too ‘sticky’ to perform fully controlled synthesis^1^.

However, along these lines, the two-dimensional confinement of a surface can undertake the task of such fingers; that is, it can be exploited for aligning the reactants. This concept initiated the new field of on-surface synthesis, which enabled forming unprecedented molecular architectures and is steadily on the rise^2–4^. In this way, the molecular structure and hence the mechanical, optical and electrical properties of low-dimensional nanoarchitectures can be controlled^5–10^. For example, graphene nanoribbons or π-conjugated polymers can be equipped with topological states^11–13^ or metallicity^14^ by introducing non-trivial structural modifications. Through chemical engineering approaches, single-molecule devices have been created on surfaces that show reversible photoswitching^15^ or tunable electroluminescence^16^ or that may be used for spintronic applications^17^.

The synthesis of such tailored materials is usually carried out on a metal surface by thermal activation, wherein the reaction selectivity and outcome are influenced by various factors, such as thermodynamics and kinetics, the activating groups of the precursors and the catalyst and template effect of the substrates. By contrast, scanning probe manipulation can avoid these obstacles by electrically triggering the chemical transformation of individual molecules via the tip of a scanning tunnelling microscopy (STM) or atomic force microscopy (AFM) instrument^18–21^. However, tip-induced intermolecular coupling is still very challenging due to the poorly controlled alignment of the molecules and the strong chemical interactions between the molecules and the metal surfaces^21^. A few successful cases were accomplished in special conditions where the molecules were constrained in a two-dimensional molecular island^22^ or at the step edges of a metal surface^23^.

**Fig. 1:**
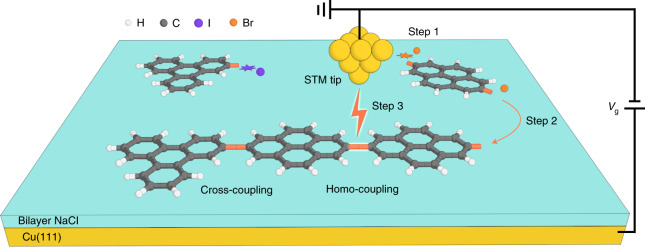
Schematic of the tip-induced block-by-block synthesis approach. Voltage pulses applied between the STM tip and the sample are used for inducing the dehalogenation of the precursors (Step 1, indicated by purple and orange stars), the transfer of the adsorbed radicals (Step 2) and the subsequent cross-/homo-coupling of the individual building blocks (Step 3). The gap voltage, *V*g, is applied to the surface, while the STM tip is grounded.

In this study, we revisit Feynman’s and Drexler’s visions and utilize the STM tip and the terraces of NaCl thin films as ‘non-fat and non-sticky fingers’ for covalently connecting different molecular building blocks via controlled manipulations (Fig. 1). The outcome of the sequential reaction steps can be conveniently followed by visual inspection with high-resolution low-temperature AFM with CO-functionalized tips^24^, which allows the identification of the chemical structures of the precursors, intermediates and products^25–33^.

As a proof of concept, two different molecular building blocks have been chosen and selectively connected controlling the chemo-, site- and regio-selectivity as well as the two-dimensional stereoselectivity. The controllability and the freedom of design are demonstrated by selective bond formation of triphenylene and pyrene building blocks. These compounds have been chosen because they are structurally similar, both possess four benzene units and they can be easily analysed by AFM. This way, the selective synthesis of triphenylene dimers in *cis* and *trans* configurations (including both *trans* mirror types) and pyrene homo-trimer as well as triphenylene/pyrene hetero-trimers via a cross-coupling reaction was achieved. The formation of *cis* and *trans* connections via thermal activation on metal substrates, however, depends strongly on the substrate material, molecular flux and reaction kinetics and is hence not fully controllable^34^. The realization of a selective cross-coupling reaction of two different partners is particularly difficult to steer even for connecting only two molecular building blocks. For example, Lewis et al. achieved a maximum selectivity of 50% towards cross-coupling by using thermally activated Ullmann coupling^35^. Additionally, other complex bonding motifs such as a carbon–iodine–carbon connection as well as fused carbon pentagons have been installed in a controlled manner.

Our approach enables the bottom-up engineering of elusive organic nanoarchitectures with atomic precision. Additionally, it allows studying the pathways of intermolecular reactions between aromatic carbon radicals by molecular manipulation. Ultimately, it paves the way for systematically studying the effects of structural modifications on the properties of organic materials, which is important for understanding and controlling molecular functionality.

## Results and discussion

In our study, 2-iodotriphenylene (IT) and 2,7-dibromopyrene (DBP) as starting materials were adsorbed on bilayer (2 ML) NaCl films supported by a Cu(111) surface. All experiments were performed with an STM/AFM instrument at 5.2 K under ultra-high vacuum (Methods). Sequential local voltage pulses were used to induce activation of the precursors (dehalogenation), movement to bring intermediates into proximity (translational and rotational manipulation of the radicals) and finally, intermolecular coupling of the building blocks. In particular, the latter two reaction steps are facilitated by the decoupling of reactive intermediates from the metal surfaces by NaCl thin films.

**Fig. 2:**
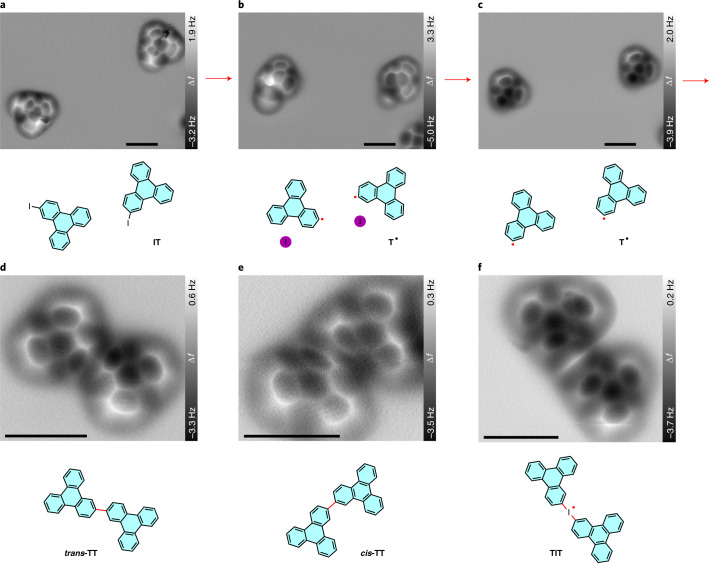
Tip-induced deiodination and intermolecular homo-coupling of IT on NaCl(2 ML)/Cu(111). **a**–**c**, Constant-height AFM frequency shift (Δ*f*) images of two pristine IT molecules (**a**) and the adsorbed T^•^ radicals before (**b**) and after (**c**) removing the adjacent iodine atoms. **d**,**e**, AFM images of a *trans* isomer (**d**) and a *cis* isomer (**e**) of **TT** produced by tip-induced homo-coupling of two T^•^ radicals. **f**, AFM image of an iodine-bridged triphenylene dimer **TIT**. Chemical structures are presented below the corresponding AFM images. The newly formed radicals and bonds are indicated by red dots and lines, respectively. The images are collected from different series. The detailed manipulation processes for the products in **d**–**f** can be found in Supplementary Figs. 10, 12 and 16, respectively. Tip–substrate distance offset Δ*z* = 120 pm (**a**), 100 pm (**b**), 110 pm (**c**), 70 pm (**d,e**) and 90 pm (**f**), relative to STM set points of 500 mV, 2 pA (**a**–**c**), 500 mV, 1.5 pA (**d**,**e**) and 500 mV, 1.3 pA (**f**). Scale bars, 1 nm for all AFM images.

First, the mono-halogenated aromatic compound IT was explored. After deposition, we observed individual molecules (Fig. 2a) and noncovalently assembled clusters of IT (Supplementary Figs. 1 and 2). The two pristine IT molecules appear in Fig. 2a as if they were bisected. This is caused by slight rotations of the intact IT around its I atom, which adsorbs close to a Na top site and acts as a pivot point for the rotation (Supplementary Fig. 3). Similar molecular movements between different adsorption positions during imaging have been observed for different compounds on different substrates^29,36,37^. The unstable adsorption also indicates a low diffusion barrier of pristine IT on NaCl(2 ML)/Cu(111), which enables its lateral manipulation (Supplementary Fig. 4) based on inelastic excitation^38,39^.

The deiodination of the IT molecules was triggered by short (10–100 ms) voltage pulses with a sample bias voltage of about 2.0 V and deactivated tip–sample feedback (Supplementary Information)^18,29,40^. Before, the metal tip was placed above the centre of the molecule with a STM set point of typically 200–500 mV and 2 pA. After deiodination, triphenylene radicals (T^•^) were generated, and the dissociated iodine atoms were usually found in their close vicinity (Fig. 2b). The iodine atoms can be deliberately removed by vertical manipulation if needed (Methods and Fig. 2c).

Unlike the chemically adsorbed radicals on metal surfaces, which possess strongly deformed adsorption conformations since their radical positions are pointing towards the surface (Supplementary Fig. 5)^29^, the radicals on NaCl(2 ML)/Cu(111) are rather planar^18^. This alignment of the activated sites in the molecular plane is favourable for the subsequent intermolecular coupling. We observed two kinds of adsorption states of the radical T^•^ that are denoted as free and bound states due to their different mobilities. The radical can be switched back and forth between these states by inelastic molecular excitations^41^ (Supplementary Figs. 6 and 7). The free state is too mobile for stable imaging, while the chemical structure of the bound state can be resolved by STM and AFM (Fig. 2c and Supplementary Fig. 6). The radicals T^•^ dominantly exist in the bound state.

In agreement with previous reports about charged states of, for example, Au atoms and pentacene molecules on NaCl layers^42,43^, the lateral and vertical shifts of the Kelvin probe parabolas that correspond to the free and bound states of the T^•^ radicals (Supplementary Fig. 7 and Supplementary Information) suggest that these are negatively charged in the bound state and neutral in the free state. However, an unambiguous identification of the charge states would require wide-range frequency shift vs. voltage [Δ*f*(*V*)] spectra (for example, −1.0 V to 2.0 V) that reveal the charging and discharging processes^43^. In our case, this could not be accomplished since the molecules shift/rotate away from the tip during the spectroscopy measurements due to their high mobility. Further details about the findings after repositioning the molecules, which are useful for rationalizing the charging and discharging processes of the T^•^ and bromopyrene (BP^•^) monoradicals, can be found in the Supplementary Information.

The controlled lateral manipulation of T^•^, which is crucial for selective coupling, was realized by voltage pulses (2.0 V, 20 ms, with metal or iodine tip) or tip–molecule forces (Supplementary Figs. 8 and 9). The tip was placed at the edge of the molecule in order to pull it towards the tip, as observed before for pentacene and 4NCuPc on NaCl(2 ML)/Cu(111) (ref. ^38^).

The intermolecular bond forming process between two adjacent T^•^ radicals was induced by one or more voltage pulses (2.0 V, 20–100 ms, metal or iodine tip). By choosing two molecules with a defined adsorption conformation (the same or the opposite on-surface handedness; Supplementary Fig. 2), we can deliberately form 2,2′-bitriphenylene (**TT**) in *trans* and *cis* configurations (Fig. 2d,e; also Supplementary Figs. 10–12 for detailed procedures), which is not fully controllable via thermally activated on-surface synthesis^34^. The two versions of *trans*-TT with opposite on-surface handedness were also selectively created (Supplementary Figs. 10 and 11). This would be an extremely difficult task for thermally activated approaches. The covalent connectivity of the products was confirmed by molecular manipulation, high-resolution STM/AFM imaging and bond-length measurements (Supplementary Figs. 13–15 and Supplementary Information).

We rationalize the formation of the new C–C bond as a result of a head-on collision of the two radical positions during the translational and rotational motions of the two adjacent molecules induced by the last voltage pulse. Please note that the voltage-pulse-induced rotations of the molecules are relatively random compared with translations. Depending on the relative orientation and distance between the two molecules, different numbers of pulses (one to ten) are required to cause the precise collision of the two radical positions and the overlap of their half-filled *sp*^2^ orbitals to form a C–C sigma bond. It is unnecessary to perfectly align the radicals in their static state and often impossible due to the preferential adsorption orientation and position of each molecule on NaCl surfaces. In our experiments, the distance between the centres of two molecules was on average 1.1 ± 0.2 nm from where a bond formation was achieved by the final voltage pulse.

Interestingly, the newly formed single C–C bond of the different **TT** molecules (red lines in Fig. 2d,e) appears somewhat darker in the AFM frequency shift images than the other C–C bonds of the triphenylene backbones. This image contrast is in good agreement with previous studies of **TT** molecules^28^, terphenyl derivates^29^ or nanographenes^44^ where single C–C bonds also appear darker than bonds within the benzene rings. Presumably this is caused by the different bond orders of the respective bonds, which is well known to influence the frequency shift contrast^32,45,46^. From this we rationalize that the lower electron density of the newly formed single C–C bond causes a lower frequency shift signal than the stronger C–C bonds inside the aromatic rings.

We also fabricated an iodine-bridged triphenylene dimer (**TIT**) featured with a C–I–C bond (Fig. 2f), which has, to the best of our knowledge, not been observed via thermally activated on-surface coupling. The bonding motif can be realized by bond formation between a T^•^ radical and a pristine IT molecule **(**Supplementary Fig. 16). As revealed by Fig. 2f, the C–I–C connection is rather symmetric. Furthermore, it is relatively stable since it survived a tip-induced rotation of the **TIT** dimer (Supplementary Fig. 16). Please note that multivalent iodine compounds have been established as stable and useful compounds in organic synthesis^47–49^. Besides the classic oxidation state of (I) there are a plethora of hypervalent iodine compounds reported with iodine in the oxidation states (III) and (V). The formation of the C–I–C bond can be rationalized by an attack of the carbon radical of a T^•^ molecule on the iodine atom of a pristine IT molecule, resulting formally in an iodine(III)-analogous structure stabilized by the surface. The linear arrangement has to be discussed in connection with the molecule–surface interactions. A detailed analysis of this hitherto-not-reported bonding motif on surfaces is the subject of ongoing studies in our laboratory.

**Fig. 3:**
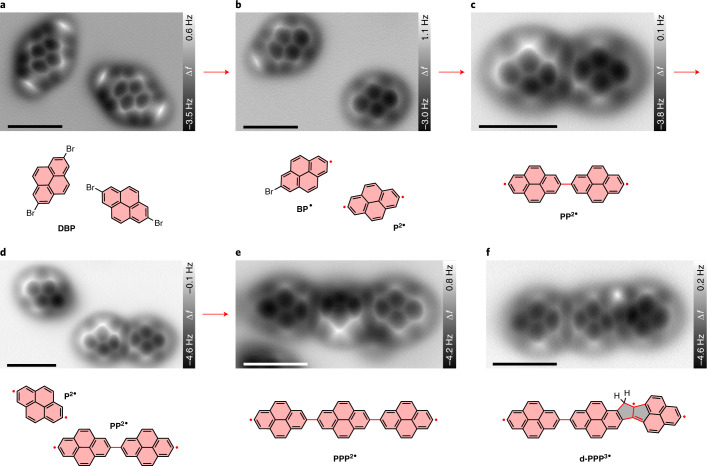
Tip-induced debrominative homo-coupling and oligomerization of DBP on NaCl(2 ML)/Cu(111). A few representative AFM images illustrate the main steps for the manipulation. **a**, Two isolated DBP molecules. **b**, A BP^•^ monoradical and a P^2•^ diradical. **c**, A PP^2•^ homo-dimer formed via homo-coupling of two pyrene diradicals. **d**, A P^2•^ diradical and a PP^2•^ homo-dimer. **e**, A PPP^2•^ homo-trimer. **f**, A defective pyrene trimer **d-PPP**^**3•**^ with two fused pentagons generated by skeletal rearrangement. Chemical structures are presented below the corresponding AFM images. The newly formed radicals and bonds are indicated by red dots and lines, respectively. The images are collected from different series. The detailed manipulation processes for the products in **e** and **f** can be found in Supplementary Figs. 21 and 22, respectively. Tip–substrate distance offset Δ*z* = 100 pm (**a**–**f**) relative to 500 mV, 2 pA. Scale bars, 1 nm for all AFM images.

Extended covalent structures can be built by utilizing multi-halogenated precursors, illustrated by DBP (Fig. 3a). The Br atoms also adsorb close to the Na top sites (Supplementary Fig. 17), similar to the adsorption position of I atoms in the case of IT molecules (Supplementary Fig. 3).

The two C–Br bonds of DBP can be cleaved sequentially by slightly different voltage pulses (2.0 V for the first and 2.1–2.2 V for the second C–Br bond). Lateral manipulation of pristine DBP molecules by voltage pulses was not achieved because a voltage higher than 2.0 V is needed. Interestingly, the voltage thresholds for the first debromination of DBP and the deiodination of IT are almost equal on NaCl(2 ML)/Cu(111) (Supplementary Fig. 18). By contrast, it requires a higher voltage to break C–Br bonds than C–I bonds on a Cu(111) surface (2.9 V versus 1.9 V; Supplementary Fig. 5) due to the higher bond energy of C–Br bonds^29^. This suggests a different excitation mechanism of the tip-induced dehalogenation on NaCl(2 ML)/Cu(111) than on metal surfaces. We postulate that this is related to the increased lifetime of the molecular negative-ion resonance due to the reduced coupling of the molecule with the metal substrate on the 2 ML NaCl films^50^.

Depending on the extent of debromination of DBP, BP^•^ and pyrene diradicals (P^2•^) were both observed (Fig. 3b). The pyrene radicals also exhibit two different adsorption states (free and bound states) between which switching was observed after applying voltage pulses (Supplementary Fig. 19). The bound state is presumably negatively charged and can be distinguished from the free state by the darker contrast (lower adsorption distance) of the radical position in STM/AFM images and the different adsorption orientation (Supplementary Fig. 19). By applying voltage pulses of 2.0 V at the radical site of BP^•^, the molecules can be pushed away from the tip along the [110] direction of the NaCl(001) surface (Supplementary Fig. 20). Pulse-induced motion of P^2•^, however, is relatively random. The directionality of the vibronically excited motion can be affected by many factors, including the polarity and the position of the voltage pulses, the intramolecular charge distribution and van der Waals tip–sample forces^38,51^.

The covalent bond between two adjacent pyrene radicals was also formed by voltage pulses (3.0 V, 10 ms, metal or CO tip). Since the main reason that a manipulated bond formation could fail is an unintended pickup of the molecules by the tip, the tip was retracted by 300 pm relative to an STM set point of 500 mV and 2 pA prior to the voltage pulses. However, the larger tip–sample distance makes a voltage of 2.0 V inefficient for triggering the manipulated lateral motion and bond formation due to the decrease of the tunnelling current and the reduced strength of the electric field. Accordingly, higher voltage pulses of 3.0 V were exploited to ensure the efficiency of the manipulation. Generally, more pulses are needed for connecting pyrene mono- and diradicals than for triphenylene radicals due to the lower mobility of pyrene radicals. The homo-coupling between two pyrene diradicals results in a covalently connected bipyrene diradical (PP^2•^; Fig. 3c). The reactive ends allow continuously extending the structure at will. In Fig. 3d, another P^2•^ diradical was brought in the vicinity of a PP^2•^ dimer, where a covalent pyrene trimer (PPP^2•^) was synthesized (Fig. 3e and Supplementary Fig. 21).

In addition to forming single C–C bonds, accessing more complex connectivities is feasible via tip-induced skeletal rearrangement^19^. We obtained a topological defect by using a higher voltage pulse of 3.5 V in the attempt to join a PP^2•^ dimer with a P^2•^ diradical (Fig. 3f and Supplementary Fig. 22). In this case, the left six-membered carbon ring of the diradical has transformed into a five-membered ring. This isomeric pyrene is annulated to the pyrene dimer via another five-membered ring. The frequency shift image in Fig. 3f reveals a rather bright feature at the CH_2_ group of the left pentagon. This is in good agreement with images of similar CH_2_ groups that were reported previously in the literature^52–54^. This example demonstrates the possibility to create fused linkages through multistep reactions, which is promising for synthesizing elusive polycyclic aromatic compounds such as low-dimensional carbon allotropes^8^.

**Fig. 4:**
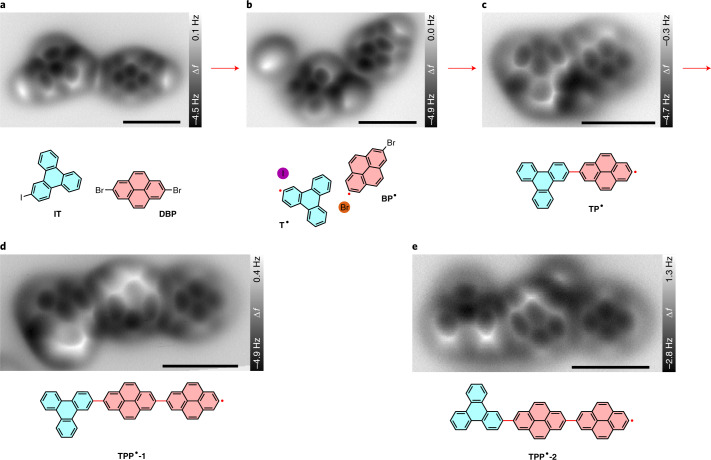
Tip-induced cross-coupling between IT and DBP on NaCl(2 ML)/Cu(111). **a**–**c**, Constant-height AFM images of an IT molecule and a neighbouring DBP molecule (**a**) and the reaction products, a T^•^ radical and a BP^•^ monoradical (**b**) and a TP^•^ hetero-dimer (**c**) generated by tip-induced dehalogenation and subsequent cross-coupling. **d**,**e**, AFM images of two TPP^•^ hetero-trimers (**TPP**^**•**^**-1** and **TPP**^**•**^**-2**) with the opposite on-surface handedness. Chemical structures are presented below the corresponding AFM images. The newly formed radicals and bonds are indicated by red dots and lines, respectively. AFM images in **d** and **e** are collected from different series than **a**–**c**. Tip–substrate distance offset Δ*z* = 90 pm (**a**) and 80 pm (**b**–**e**) relative to 500 mV, 2 pA. Scale bar, 1 nm for all the AFM images.

Ultimately, we demonstrate the capability of connecting different molecular building blocks in a controlled manner, which is a challenging task via conventional thermal coupling approaches because homo-coupling may prevail^35^. Furthermore, the ability to covalently assemble different types of organic molecules provides opportunities for systematic studies of molecular heterojunctions^55,56^. We use the cross-coupling reaction between IT and DBP as an example to illustrate the high controllability of the tip-induced reactions between two molecules endowed with different molecular backbones and activating groups. Similar to the above homo-couplings, the pristine IT and DBP molecules (Fig. 4a) were first dehalogenated (Fig. 4b) and subsequently fused to form a covalent TP^•^ hetero-dimer (Fig. 4c) via voltage pulses. In case the two molecules are farther apart from each other, lateral manipulation is needed before initiating the intermolecular coupling (Supplementary Fig. 23).

As aforementioned, the covalent assembly is extendable since there is a carbon radical remaining at one end of the TP^•^ dimer. The structure can be either terminated by attaching another monoradical (Fig. 2d,e) or extended further by attaching another multi-halogenated precursor. Here a pyrene diradical is coupled with the dimer, generating a **TPP**^**2•**^ trimer (Fig. 4d). Similarly, the enantiomeric **TPP**^**2•**^ trimer was fabricated by choosing an IT molecule with the opposite on-surface handedness as a precursor (Fig. 4e and Supplementary Fig. 24), realizing an asymmetric transformation to a specific enantiomer with planar chirality. The covalent connectivity of the trimers was confirmed by molecular manipulations, high-resolution STM/AFM imaging and bond-length measurements (Supplementary Figs. 21, 25 and 26).

The presented results demonstrate that scanning probe manipulation is a powerful tool for the selective covalent assembly of organic building blocks. In this way, covalent nanoarchitectures that hitherto remained elusive become accessible. The approach also opens ways for studying structural modifications of tailored molecules and intermolecular reaction pathways on insulating substrates.

## Methods

### Source of the molecules

DBP (purity >97%) was purchased from the TCI company. The synthesis of IT has been reported in our recent paper^57^.

### Sample preparation

The Cu(111) crystal (Mateck) was cleaned by repeated sputtering and annealing processes. NaCl (purity >99%, Sigma Aldrich) was heated to 823 K and deposited onto a clean Cu(111) surface held at 273–282 K for 5 min, leading to the surface being partially covered by 2 ML NaCl(001) islands. We then dosed a submonolayer of IT and DBP molecules onto the cold (~6 K) NaCl(2 ML)/Cu(111) surface using a commercial evaporator (Kentax). We also dosed CO molecules to the sample surface for tip modification.

### STM and AFM measurements

All the STM and AFM measurements were performed at ~5.2 K under ultra-high-vacuum conditions (base pressure <1.0 × 10^−10^ mbar) using a commercial STM/AFM system (Scienta Omicron). The STM tip was grounded, and all the voltages mentioned in this paper refer to the sample bias voltage. We used a qPlus-type force sensor^58^ with a resonance frequency of *f* ≈ 26.98 kHz, an oscillation amplitude of *A* ≈ 56–71 pm and a quality factor of *Q* ≈ 10,000–50,000. AFM imaging was carried out in frequency modulation mode with a constant oscillation amplitude using external phase-locked loop electronics (MFLI, Zürich Instruments). A small sample bias voltage (submillivolt) was applied during constant-height AFM imaging to minimize the tunnelling current. The positive and negative signs of the tip–substrate distance offset Δ*z* represent the increase and decrease of the tip–substrate distance with respect to a certain STM set point, respectively. CO-terminated tips were exploited for chemical bond resolution. To obtain a CO-terminated tip, a metal tip was first sharpened by several voltage pulses and controlled indentations into the Cu(111) surface, and then the tip was vertically pressed against a CO molecule adsorbed on the NaCl(2 ML)/Cu(111) surface to pick it up. Therefore, the tip was first placed above the CO with an STM set point (typically 500 mV and 2 pA) and then moved towards the CO molecule until a sudden jump in the current signal was observed. The pickup was confirmed by the enhanced contrast of the subsequent STM and AFM images. Iodine atoms were vertically manipulated in a similar way.

### Tip-induced dehalogenation

The dehalogenation, manipulation and intermolecular coupling were all induced by voltage pulses (except Supplementary Fig. 9). The deiodination of IT and the first debromination of DBP were effectively triggered by short voltage pulses of +2.0 V (sample bias) with a duration of 10–100 ms at tip heights determined by the STM set points (typically 200–500 mV, 2 pA)^59^. This voltage was found to be also sufficient to induce the subsequent lateral motion and intermolecular coupling. For the second debromination of DBP, a slightly higher voltage (2.1–2.2 V) was needed. The tip was usually placed above the centre of the molecules to induce the cleavage of the halogen. For this process we mostly used metal tips. In cases where we needed to remove the halogens from the surface, we picked them up by vertically pressing a metal tip against them (as described in the paragraph above).

### Tip-induced molecular translations and rotations

The translational and rotational motions of the dehalogenated radicals were triggered with similar parameters as those used for dehalogenation. Voltages lower than 2.0 V usually failed to move the molecular radicals. Presumably, this is related to the probability of electron injection into the molecular frontier orbitals. The tip was placed at the edge of the molecules to intentionally push/pull them in a preferential direction. Metal tips were often used, while similar behaviours of manipulation were observed for CO and I tips.

### Tip-induced intermolecular coupling

The short voltage pulses mentioned above can also be used to trigger the final step of tip-induced intermolecular coupling. Alternatively, a safer approach was frequently used to avoid unintentional vertical manipulation (pickup) of the molecules. Therefore, the STM tip (metal or CO tip) was first positioned above one of the two adjacent molecules with constant-current feedback (typically 500 mV, 2 pA). Then the feedback was switched off and the tip was lifted away from the sample by 300 pm. Finally, a short voltage pulse of 3.0 V and 10 ms was applied between the tip and the sample, which led to either the covalent coupling or just the repositioning of the molecules below the tip.

## Online content

Any methods, additional references, Nature Research reporting summaries, source data, extended data, supplementary information, acknowledgements, peer review information; details of author contributions and competing interests; and statements of data and code availability are available at 10.1038/s41557-021-00773-4.

### Supplementary information

Supplementary Information Supplementary Figs. 1–26, text and refs. 1–4.

## Data Availability

All data is available in the main text or the Supplementary Information.

## 2-bromopyren-7-yl

PubChemID: 441658051
InChIKey: CQBRKYKJAWZNNQ-UHFFFAOYSA-N
MDL Molfile
Chemdraw file

## *cis*-2,2'-bitriphenylene

PubChemID: 441658040
InChIKey: VSULMNMDXCGMFW-UHFFFAOYSA-N
MDL Molfile
Chemdraw file

## 2,7-dibromopyrene

PubChemID: 441658041
InChIKey: IGTQPXMEWQTTBJ-UHFFFAOYSA-N
MDL Molfile
Chemdraw file

## 12-(pyren-2,7-diyl)-16,16a-dihydrophenaleno[1',9':4,5,6]pentaleno[2,1-*a*]pyren-4,16a-diyl

PubChemID: 441658042
InChIKey: YHCSJGURXJXAPC-UHFFFAOYSA-N
MDL Molfile
Chemdraw file

## 2-iodotriphenylene

PubChemID: 441658043
InChIKey: ZMZNYRZSJDYTIO-UHFFFAOYSA-N
MDL Molfile
Chemdraw file

## pyren-2,7-diyl

PubChemID: 441658044
InChIKey: SIZNXVDSKSJVFR-UHFFFAOYSA-N
MDL Molfile
Chemdraw file

## 2,2'-bipyren-7,7'-diyl

PubChemID: 441658045
InChIKey: UHPIOJPWDJOTBK-UHFFFAOYSA-N
MDL Molfile
Chemdraw file

## 2,2':7',2''-terpyren-7,7''-diyl

PubChemID: 441658046
InChIKey: SKAKQWPORNQHOG-UHFFFAOYSA-N
MDL Molfile
Chemdraw file

## 7'-(triphenylen-2-yl)-2,2'-bipyren-7-yl

PubChemID: 441658047
InChIKey: SJGCJPNDPOLWBI-UHFFFAOYSA-N
MDL Molfile
Chemdraw file

## 7-(triphenylen-2-yl)-pyren-2-yl

PubChemID: 441658048
InChIKey: LCTFKLFAFBRTSR-UHFFFAOYSA-N
MDL Molfile
Chemdraw file

## triphenylen-2-yl

PubChemID: 441658049
InChIKey: ILTPMPRPHHZIGO-UHFFFAOYSA-N
MDL Molfile
Chemdraw file

## *trans*-2,2'-bitriphenylene

PubChemID: 441658050
InChIKey: VSULMNMDXCGMFW-UHFFFAOYSA-N
MDL Molfile
Chemdraw file
