# Supplementary material for: Constructing covalent organic nanoarchitectures molecule by molecule via scanning probe manipulation
Source: Nat Chem. 2021 Sep 2;13(11):1133–9. doi: 10.1038/s41557-021-00773-4 (PMC8550974; doi:10.1038/s41557-021-00773-4)
Supplement: Supplementary file 1 — Supplementary Figs. 1–26, text and refs. 1–4. [file 41557_2021_773_MOESM25_ESM.pdf]

nature portfolio

https://doi.org/10.1038/s41557-021-00773-4

Supplementary information

Constructing covalent organic nanoarchitectures molecule by molecule via scanning probe manipulation

In the format provided by the authors and unedited

Supplementary Information for

Constructing covalent organic nanoarchitectures molecule by molecule via scanning probe manipulation

Authors: Qigang Zhong[1,2]*, Alexander Ihle[1,2], Sebastian Ahles[2,3], Hermann A. Wegner[2,3], Andre Schirmeisen[1,2]*, Daniel Ebeling[1,2]*

Affiliations:
[1]Institute of Applied Physics (IAP), Justus Liebig University Giessen, Heinrich-Buff-Ring 16, 35392 Giessen, Germany

[2]Center for Materials Research (LaMa), Justus Liebig University Giessen, Heinrich-Buff-Ring 16, 35392 Giessen, Germany

[3]Institute of Organic Chemistry, Justus Liebig University Giessen, Heinrich-Buff-Ring 17, 35392 Giessen, Germany

*Correspondence to: Qigang.Zhong@ap.physik.uni-giessen.de; Andre.Schirmeisen@ap.physik.uni-giessen.de; Daniel.Ebeling@ap.physik.uni-giessen.de

This PDF file includes:

Supplementary Text
Supplementary Figures 1–26
Supplementary references 1–4

Supplementary Text

Voltage thresholds for dehalogenation on NaCl(2ML)/Cu(111) and on Cu(111).

The voltage thresholds for dehalogenation on NaCl(2ML)/Cu(111) were measured in the following steps: First, a Cu tip was positioned over the center of an **IT** or a **DBP** molecule with an STM set point of 500 mV and 2 pA. Second, the STM feedback was deactivated and the tip was lifted up by 300 pm. Third, the sample bias voltage was ramped up from 1.5 V to 2.3 V with a speed of 0.016 V/s and a current versus voltage $I(V)$ curve was simultaneously recorded. The voltage ramp was stopped when a sudden jump in the $I(V)$ spectrum was observed. We then took an STM image to confirm the successful dehalogenation. In total, seven **IT** molecules and ten **DBP** molecules were tested with this procedure. The dehalogenation was successful in all these cases. For comparability, the same metal tip was used for both the deiodination of **IT** and the debromination of **DBP** (Supplementary Fig. 18). The average voltage thresholds for the deiodination of **IT** and the first debromination of **DBP** were measured to be (2.14 ± 0.11) V and (2.16 ± 0.06) V, respectively (Supplementary Fig. 18). Please note, in all other presented experiments, we simply used short voltage pulses of 2.0–2.2 V and 10–100 ms for triggering the debromitation and deiodination. In the majority of cases such pulses directly lead to the dehalogenation. However, the molecules can also be moved laterally and/or rotated. In such case we repeatedly apply voltage pulses until the dehalogenation happens. The tip identity (e.g., CO modified tip) seems to play a minor role for the dehalogenation process.

A similar method was used to measure the voltage thresholds for voltage pulse-induced dehalogenation on Cu(111). A CO tip was positioned over the center of an **IT** molecule with an STM set point of 100 mV and 10 pA. Then, we switched off the STM feedback and lifted up the tip by 200 pm. Finally, the sample voltage was ramped up from 1.5 V until a sudden change in the current channel occurred. A voltage of about 1.9 V led to the deiodination of the **IT** molecule (Supplementary Fig. 5). However, the voltage threshold of the first debromination of **DBP** is difficult to measure with this method because a voltage ramp often induced the lateral motion of **DBP** molecules before debromination. Instead, we triggered the first debromination by directly applying voltage pulses (2.9 V, 100 ms) above the C–Br bonds at a tip-height determined by the tunneling set point (100 mV, 10 pA). To cleave the second C–Br bond, the same method for deiodination was used and a voltage threshold of about 3.1 V was measured (Supplementary Fig. 5).

Charge states of free and bound radicals.

As reported in the literature the charge states of molecules can be distinguished by using Kelvin probe force spectroscopy (KPFS), which measures the local contact potential difference (LCPD) between the tip and the sample[1]. Differently charged states of the molecules change the LCPD, which shifts the $\Delta f(V)$ parabolas vertically and laterally with respect to that of the neutral state. To perform a KPFS measurement, the tip was first positioned above the radical site of a molecule with an STM set point (e.g., 500 mV, 2pA); Then the STM feedback was deactivated and the tip was lifted up by 200 pm; Finally, a $\Delta f(V)$ spectrum was recorded.

The lateral and vertical shift of the $\Delta f(V)$ parabolas suggests that the bound states of the **T$^\bullet$** radical (Supplementary Fig. 7) and the **BP$^\bullet$** monoradical (Supplementary Fig. 19) should be both negatively charged[1], while the free states should be neutral. Due to their high mobility the molecules tend to shift/rotate away from the tip at high negative and positive sample bias voltages (< −0.5 V and > 1.5 V), which prevents from the record of a wide-range (−1.0 V to 2.0 V) $\Delta f(V)$ or $I(V)$ spectrum.

Please note that molecules are dominantly found in their bound state (presumably negatively charged). In case a molecule in the bound state was repositioned for a voltage > 1.5 V it was found either in the same state or in the free state (presumably neutral). We rationalize that the electron can tunnel from the molecule into the Cu(111) substrate during the lateral movement of the molecule since we use an

NaCl film of only 2ML thickness. In case a molecule in the bound state was repositioned by a voltage of < −0.5 V it usually changed to the free state, which would imply a discharging process, i.e., an electron transfer from the molecule into the tip.

In case a free (presumably neutral) molecule was repositioned for a voltage > 1.5 V it was afterwards often found in the bound state (presumably negative). This would imply a charging process i.e., an electron transfer from the tip into the molecule.

Bond length measurements.
Bond-resolved AFM images often encounter distortions due to the flexibility of the CO molecule at the tip apex, making it difficult to determine reliable bond lengths[2-4]. To measure the length of the newly formed C–C bonds between molecules, we first fitted the chemical structures of the monomers onto the AFM images, and then took the distance between two connected carbon atoms as the measured bond length (see red lines in Supplementary Figs. 15 and 26). The measured bond lengths are between 1.21–1.62 Å which are close to the length of sp^2–sp^2 C–C bonds (about 1.47 Å).

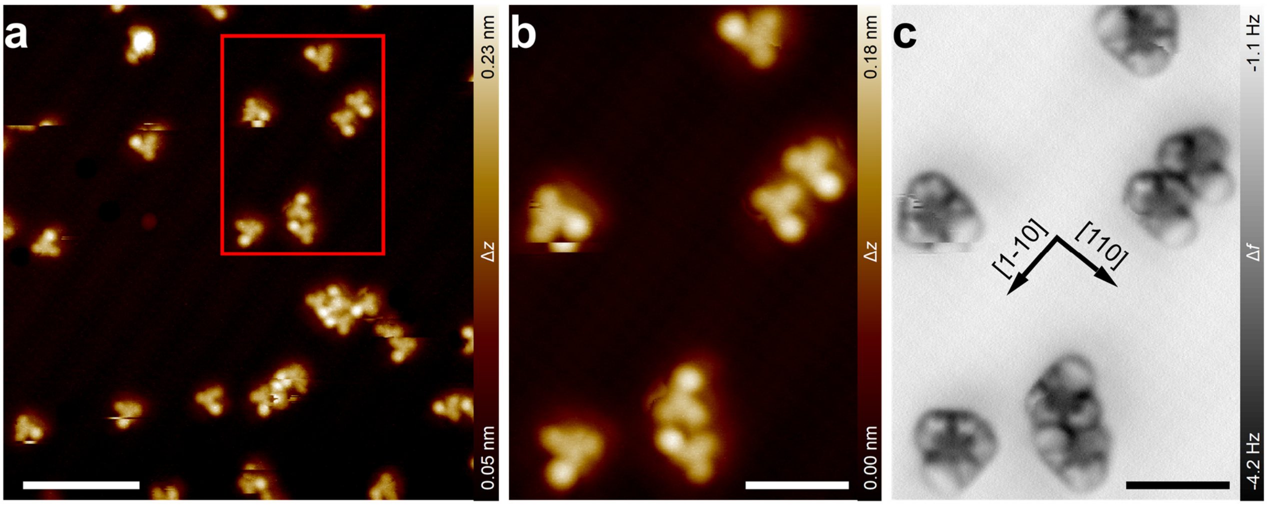

Supplementary Figure 1 | Pristine IT molecules on NaCl(2ML)/Cu(111). a, STM overview after *in-situ* deposition of **IT** molecules onto a cold (about 6 K) NaCl(2ML)/Cu(111) surface. **b**, Zoom-in STM image of the area marked with a red rectangle in **a**. **c**, Constant-current AFM frequency shift image recorded simultaneously with **b**. Imaging parameters: (**a**–**c**) 500mV, 1.5 pA. Scale bars, 5 nm (**a**) and 2 nm (**b**,**c**).

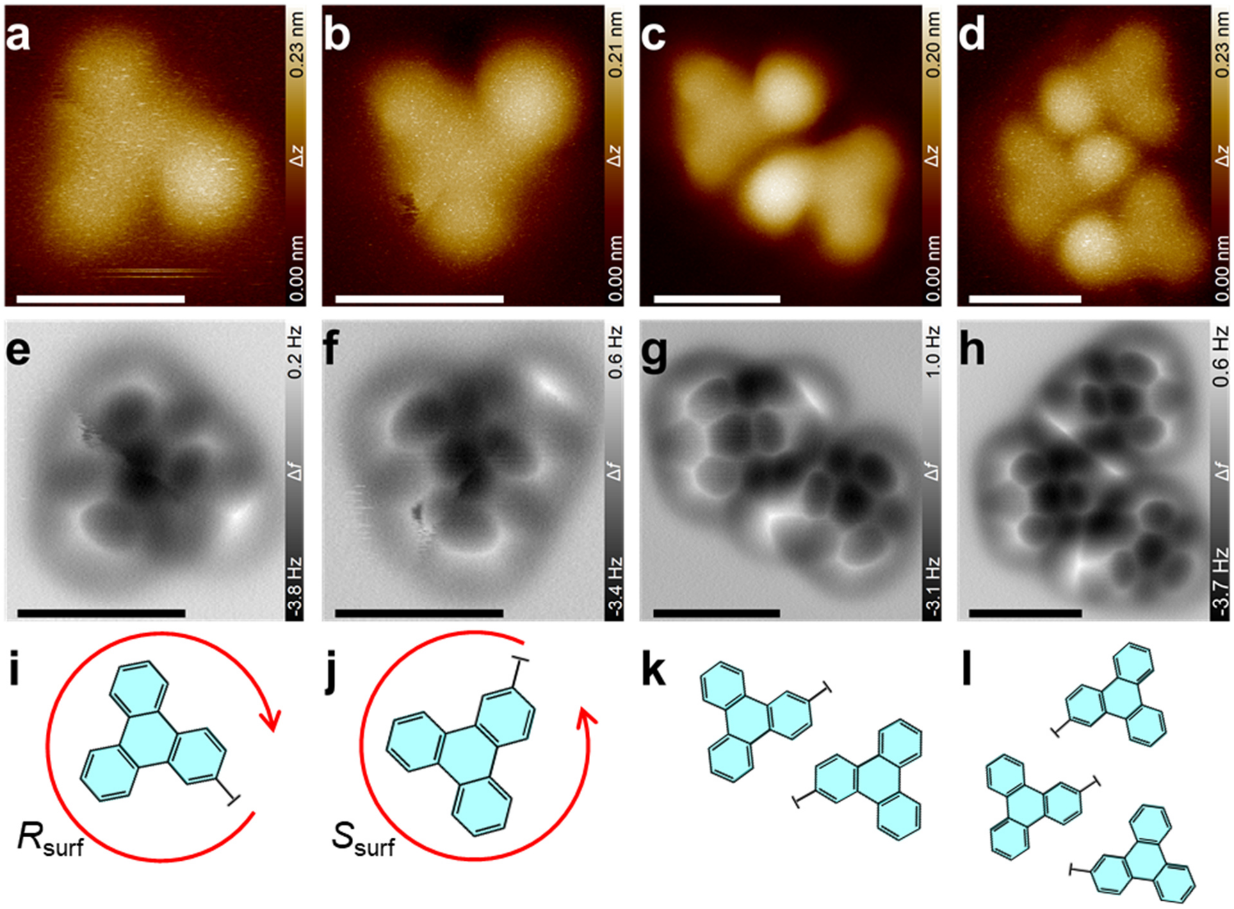

Supplementary Figure 2 | High-resolution STM and AFM images of single IT molecules and small molecular clusters on NaCl(2ML)/Cu(111). a–d, STM images of a "righthanded" R_{surf} (**a**) and a "lefthanded" S_{surf} (**b**) **IT** molecule, a noncovalent **IT** dimer (**c**) and a noncovalent **IT** trimer (**d**). **e**–**l**, Constant-height AFM images (**e**–**h**) and chemical structures (**i**–**l**) corresponding to **a**–**d**, respectively. Imaging parameters: (**a**,**b**) 600 mV, 1.3 pA. (**c**,**d**) 500 mV, 1.3 pA. Tip height offset Δz = 80 pm (**e**,**g**,**h**) and 90 pm (**f**), relative to 500 mV, 1.3 pA. Scale bar, 1 nm.

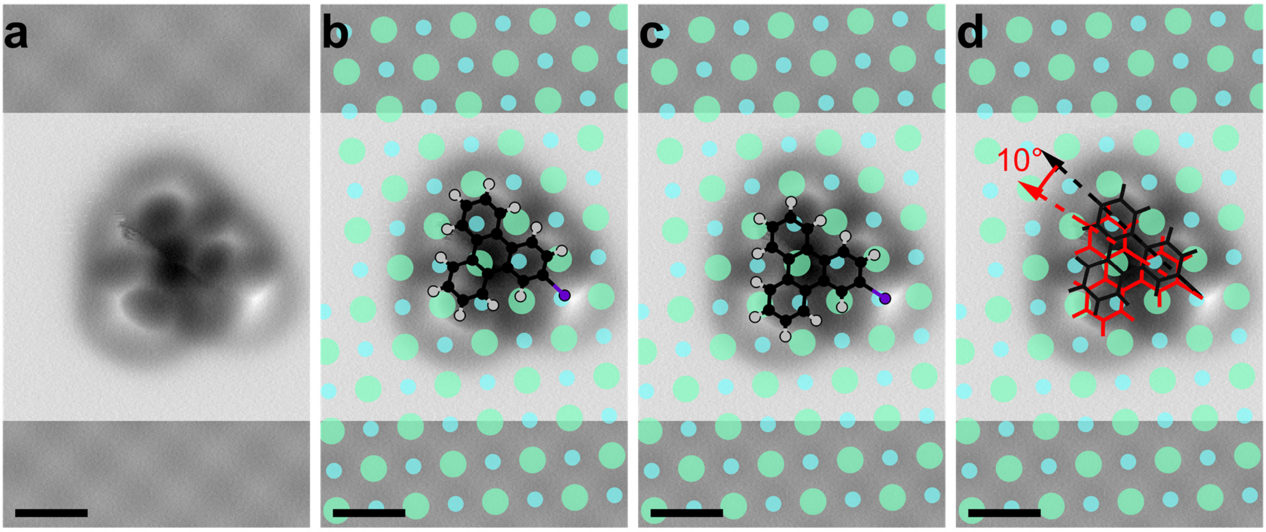

Supplementary Figure 3 | Adsorption position and orientation of isolated IT molecules on NaCl(2ML)/Cu(111). Isolated **IT** molecules exhibit bistable adsorption structures on NaCl(2ML)/Cu(111). **a**, Segmented constant-height AFM image with atomic resolution of both the NaCl surface (top and bottom) and the **IT** molecule (middle). **b**,**c**, The same AFM image as **a** on which a NaCl lattice and a molecular model are superimposed. The molecular model of **IT** is fitted to the two different adsorption positions separately. Elements are color-coded: Cl (light green), Na (light blue), C (black), H (grey), I (purple). **d**, A combination of the fitted **IT** structures in **b** and **c** revealing an angular rotation of 10° around the iodine atom, which acts as a pivot point. Tip–substrate distance offset $\Delta z = -140$ pm (for the NaCl surface) and 80 pm (for the molecule) relative to 500 mV, 1.3 pA. Scale bar, 0.5 nm.

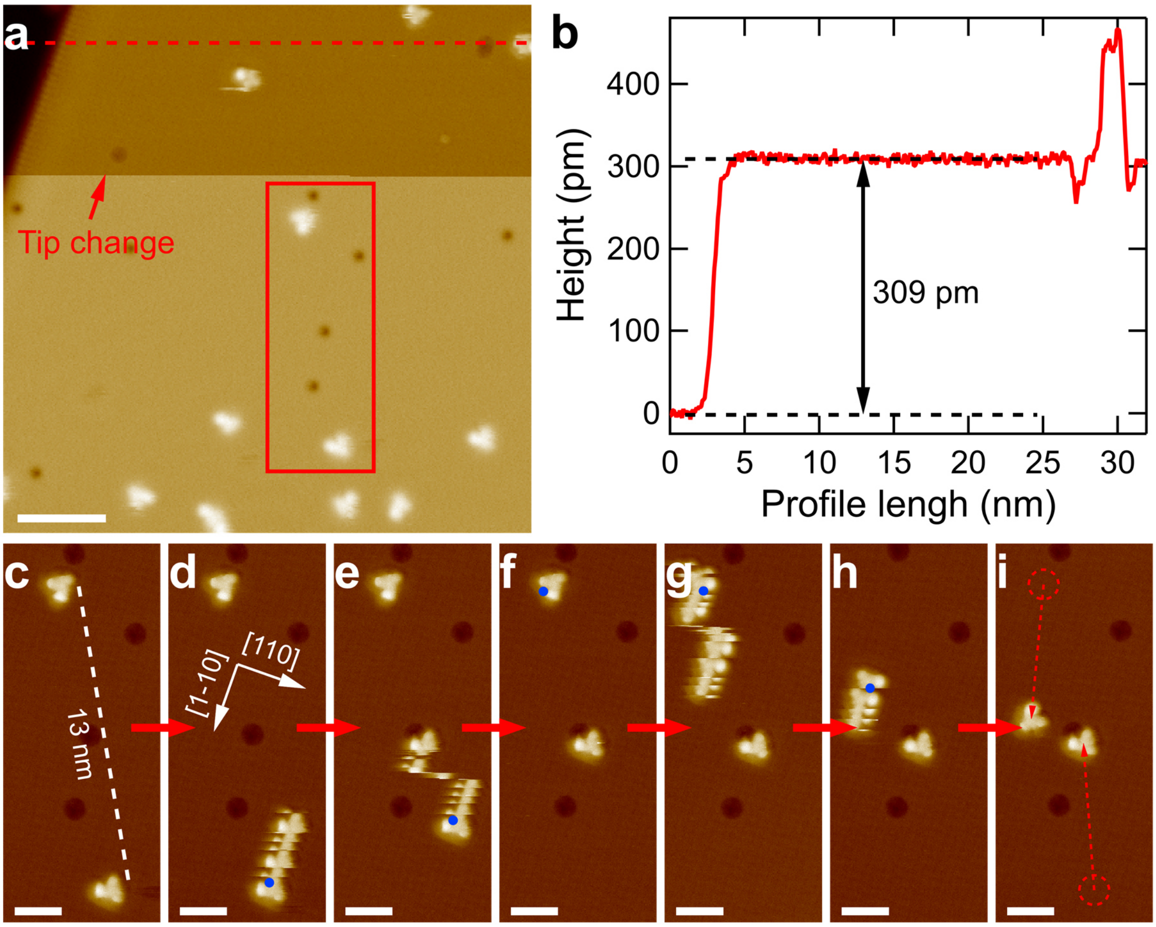

Supplementary Figure 4 | Voltage-pulse-induced repositioning of single IT molecules on NaCl(2ML)/Cu(111). The blue dot in an STM image marks the position of the voltage pulses, and the result is shown in the next STM image. **a**, STM overview of a NaCl(2ML)/Cu(111) surface decorated with isolated **IT** molecules. **b**, Line profile along the red dashed line in (A) across a step edge of the bilayer NaCl island. **c**–**i**, A series of STM images showing the lateral manipulation process of the two intact **IT** molecules within the red rectangle in **a**. The molecular movements were driven by short voltage pulses (CO tip, 1.5 V, 10 ms). In **d**,**e**,**g**,**h** only the position of the first pulse is marked. The total displacement of the two **IT** molecules with respect to the initial positions (red dashed circles) is indicated with red dashed arrows in **i**. Imaging parameters: 500 mV, 2 pA for all the STM images. Scale bars, 5 nm (**a**) and 2 nm (**c-i**).

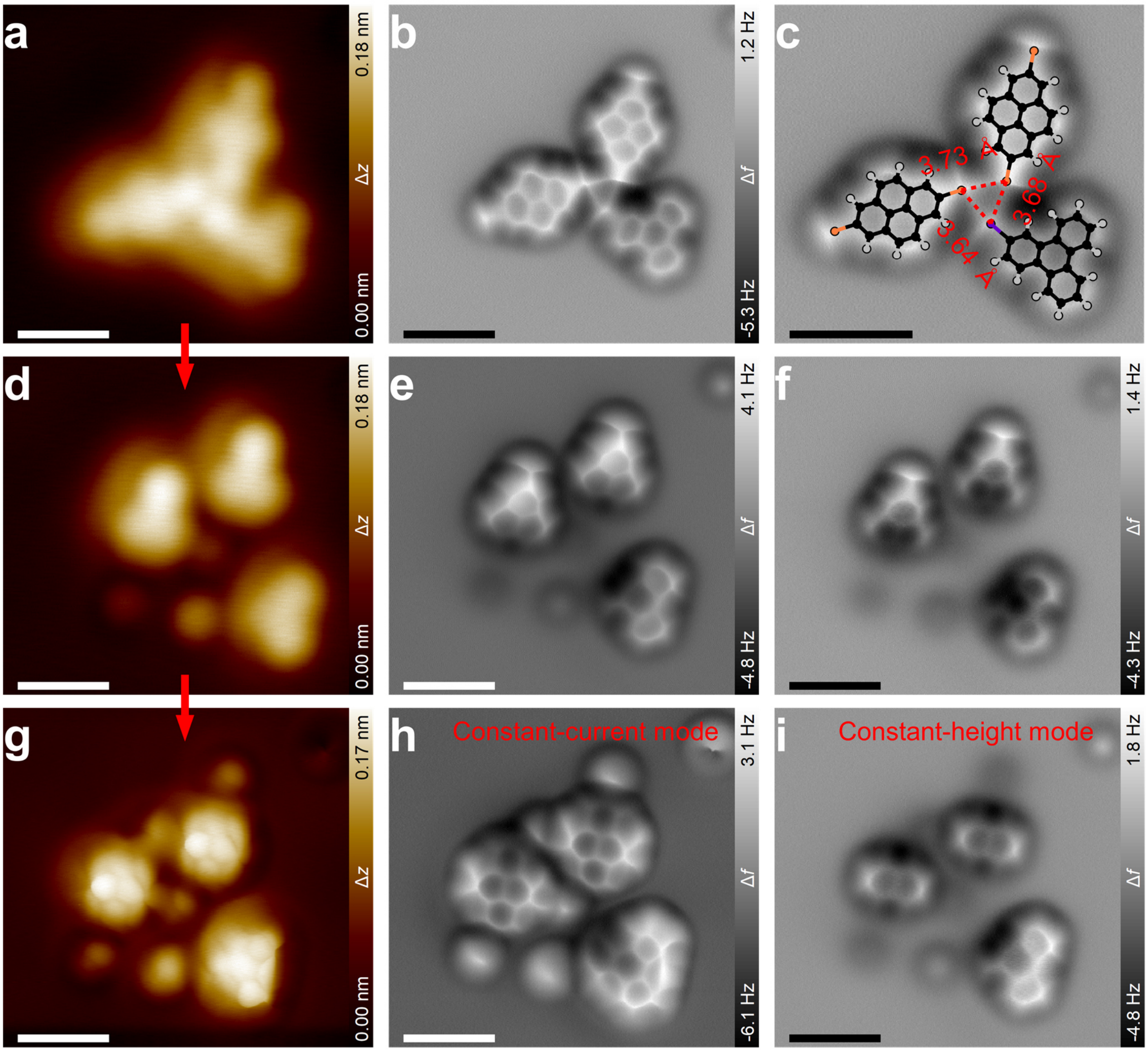

Supplementary Figure 5 | Tip-induced deiodination of IT and debromination of DBP on Cu(111). a,**b**, STM image (**a**) and constant-height AFM image (**b**) of a halogen-bonded cluster consisting of two **DBP** molecules and one **IT** molecule. **c**, Same AFM image as **b** on which molecular models were superimposed. The lengths of the halogen bonds were indicated on the image. **d**–**f**, STM (**d**) and constant-height AFM (**e**,**f**) images after deiodination (1.9 V, $\Delta z = 200$ pm, CO tip) of the **IT** molecule and partial debromination (2.9 V, 100 ms, CO tip) of the two **DBP** molecules. **g**–**i**, Constant-current STM (**g**) and AFM (**h**) images and constant-height AFM image (**i**) after cleaving the second C–Br bond (3.1 V, $\Delta z = 200$ pm, CO tip) of the two **DBP** molecules. Imaging parameters: (**a**,**d**) 100 mV, 10 pA. (**g**,**h**) 10 mV, 100 pA. Tip height offset $\Delta z = -70$ pm (**b**,**c**,**e**,**i**) and −40 pm (**f**) relative to 100 mV, 10 pA. Scale bar, 1 nm.

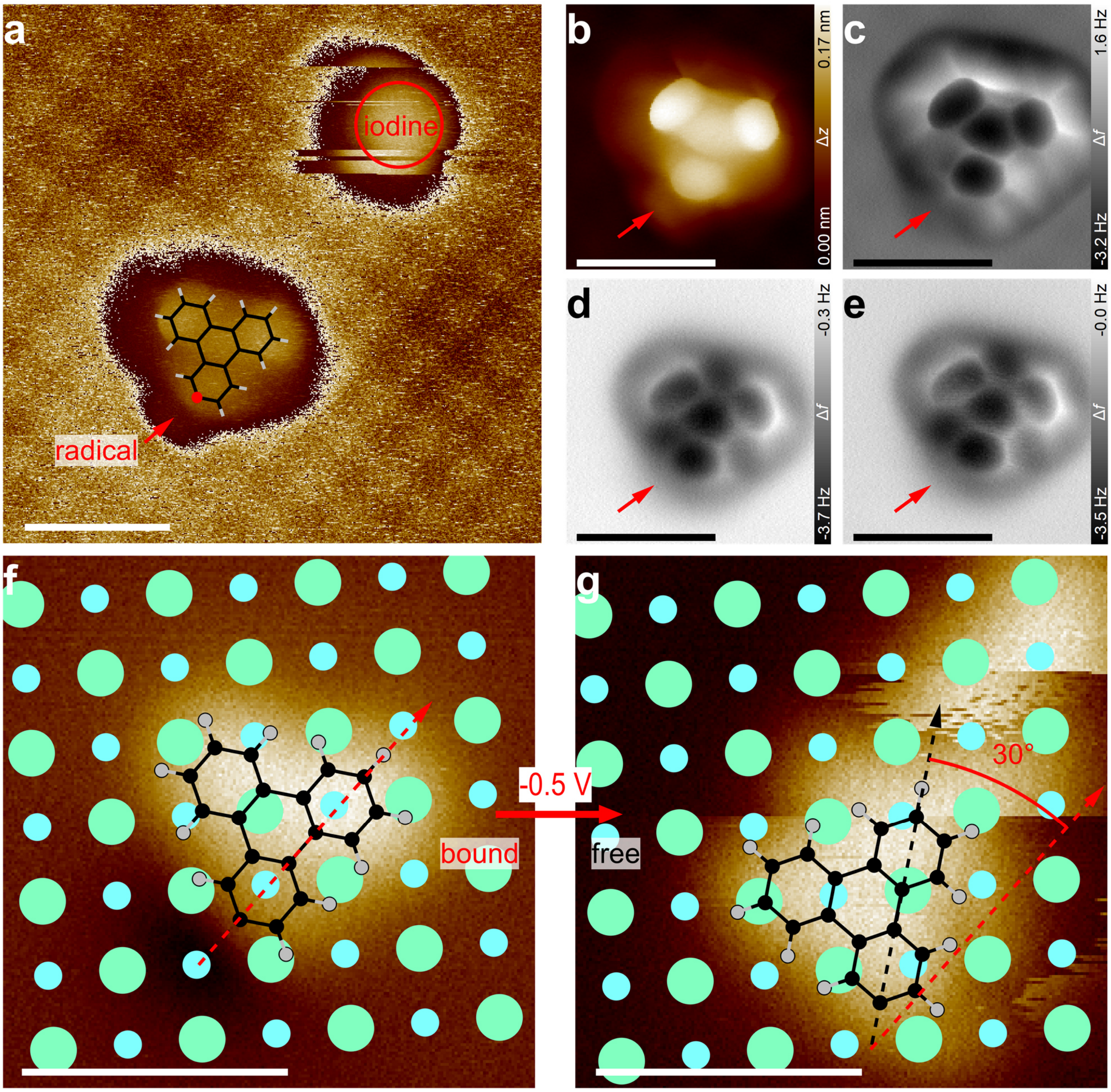

Supplementary Figure 6 | High-resolution imaging of a single T$^{•}$ radical in bound and free states on NaCl(2ML)/Cu(111). a, Atomically resolved STM image of a **T$^{•}$** radical in bound state. Image contrast is separately adjusted for the surface and the molecule. **b**,**c**, Simultaneously recorded constant-current STM (**b**) and AFM (**c**) images of the **T$^{•}$** radical. **d**,**e**, Constant-height AFM images of the **T$^{•}$** radical at different tip heights. The red arrow in **a**–**e** denotes the radical site. **f**,**g**, STM images of the **T$^{•}$** radical in bound and free states, respectively. The transition from the bound state to the free state was triggered by a voltage pulse of −0.5 V and 100 ms. The NaCl lattice and molecular model superimposed on the images reveal the different adsorption positions and an angular offset of 30° between the two states. Elements are color-coded: Cl (light green), Na (light blue), C (black), H (grey). Imaging parameters: (**a**) 300 mV, 1.3 pA. (**b**,**c**) 300 mV, 2 pA. Tip height offset Δz = 80 pm (**d**) and 70 pm (**e**) relative to 500 mV, 1.3 pA. (**f**) 200 mV, 2 pA. (**g**) 500 mV, 2 pA. Scale bar, 1 nm.

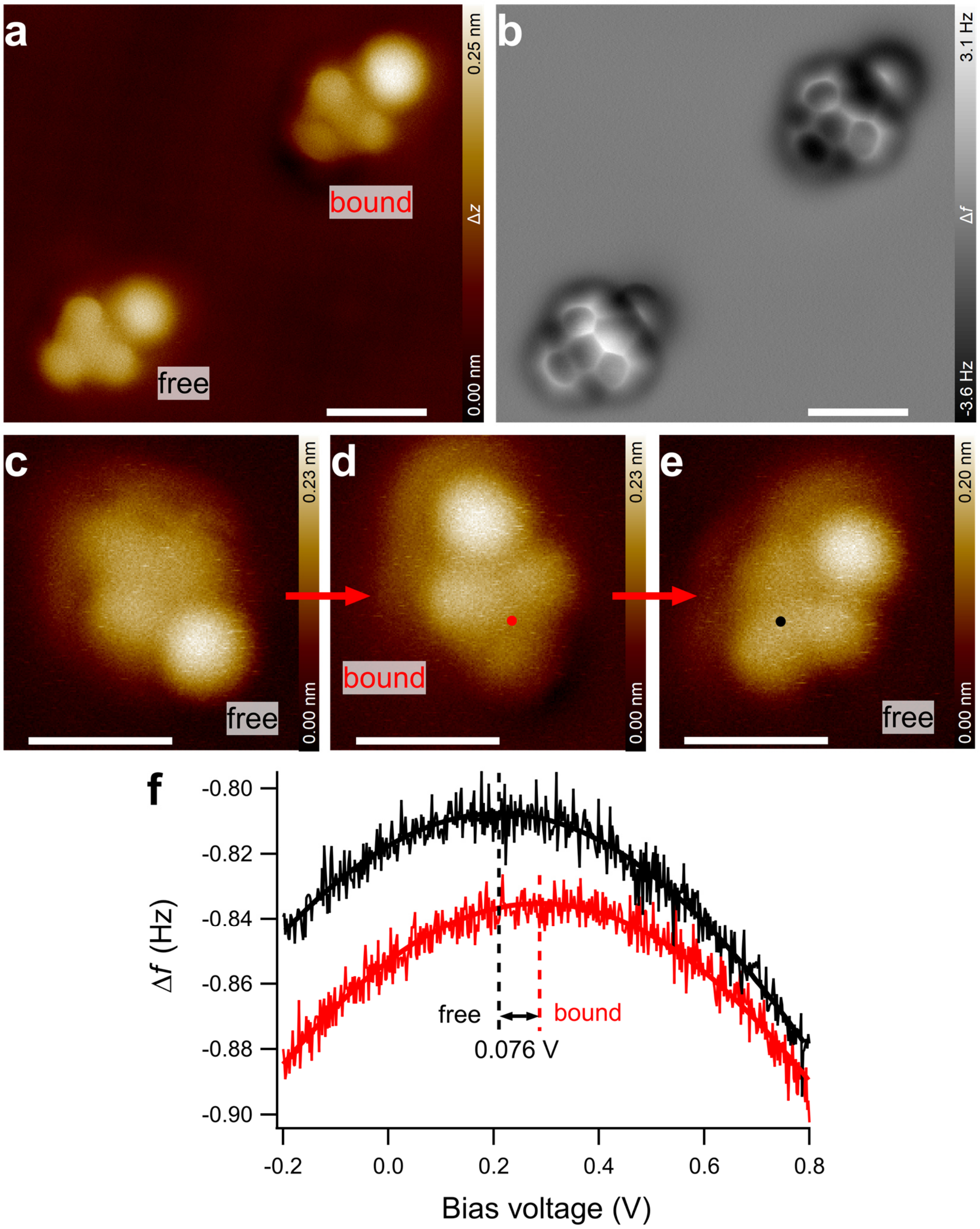

Supplementary Figure 7 | High-resolution imaging and KPFS measurements of iodine-anchored T• radicals in bound and free states on NaCl(2ML)/Cu(111). a, STM image of two **T•** radicals in bound and free states, respectively. Both molecules are anchored by their dissociated iodine atoms for stable imaging. **b**, Constant-height AFM image of the two molecules. **c**–**e**, STM images of an iodine-stabilized **T•** radical. The **T•** radical was switched reversibly between the free and bound state by voltage pulses of 2.0 V and 20 ms with a CO tip. **f**, KPFS spectra recorded with a CO tip at the center of the **T•** radical as marked in **d**,**e** with red and black dots for the bound and free states, respectively. Imaging parameters: (**a**) 500 mV, 2 pA. (**b**) Tip height offset $\Delta z = -90$ pm relative to 500 mV, 2 pA. (**c**–**e**) 500 mV, 1.5 pA. Scale bar, 1 nm.

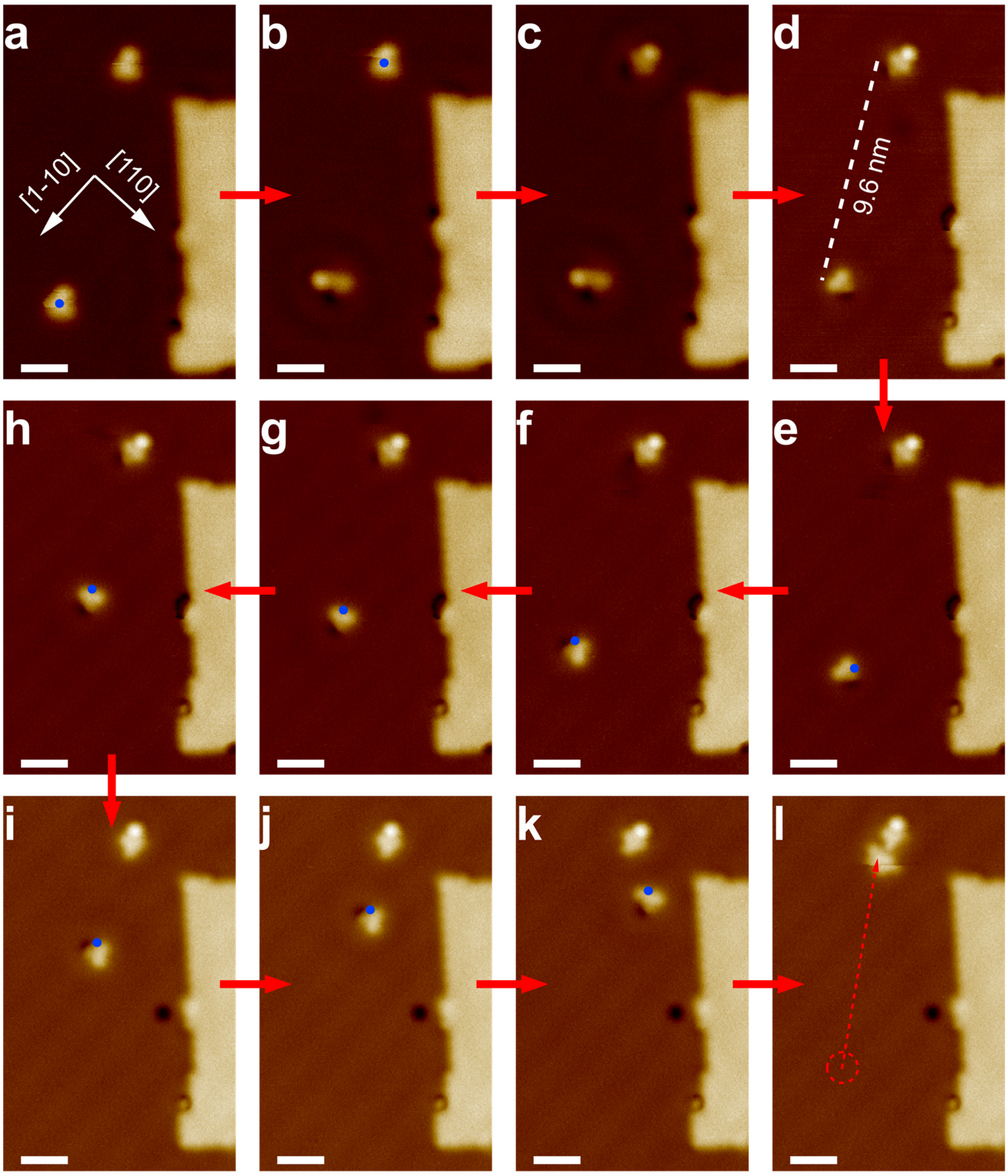

Supplementary Figure 8 | Voltage-pulse-induced repositioning of a single T• radical on NaCl(2ML)/Cu(111). The blue dot in an STM image marks the position of the voltage pulses, and the result is shown in the next STM image. **a**–**c**, STM images showing two intact **IT** molecules (**a**) and the deiodination of the bottom (**b**) and the top (**c**) molecules by voltage pulses of 2.0 V. **d**, The iodine atom next to the bottom **T•** radical was picked up by the tip. **e**–**l**, A series of STM images recording the directional manipulation of the **T•** radical towards the top molecule pulled by the I tip via applying short voltage pulses (2.0 V, 20 ms) at the edge of the molecule. One or a few pulses were applied between two frames. The manipulated **T•** radical was in the bound state featured with the dark depression at the radical site. The total displacement of the bottom **T•** radical with respect to the initial position (red dashed circle) is indicated with a red dashed arrow in (**l**). Imaging parameters: (**a**–**c**) 200 mV, 2 pA. (**d**–**l**) 500 mV, 2 pA. Scale bar, 2 nm.

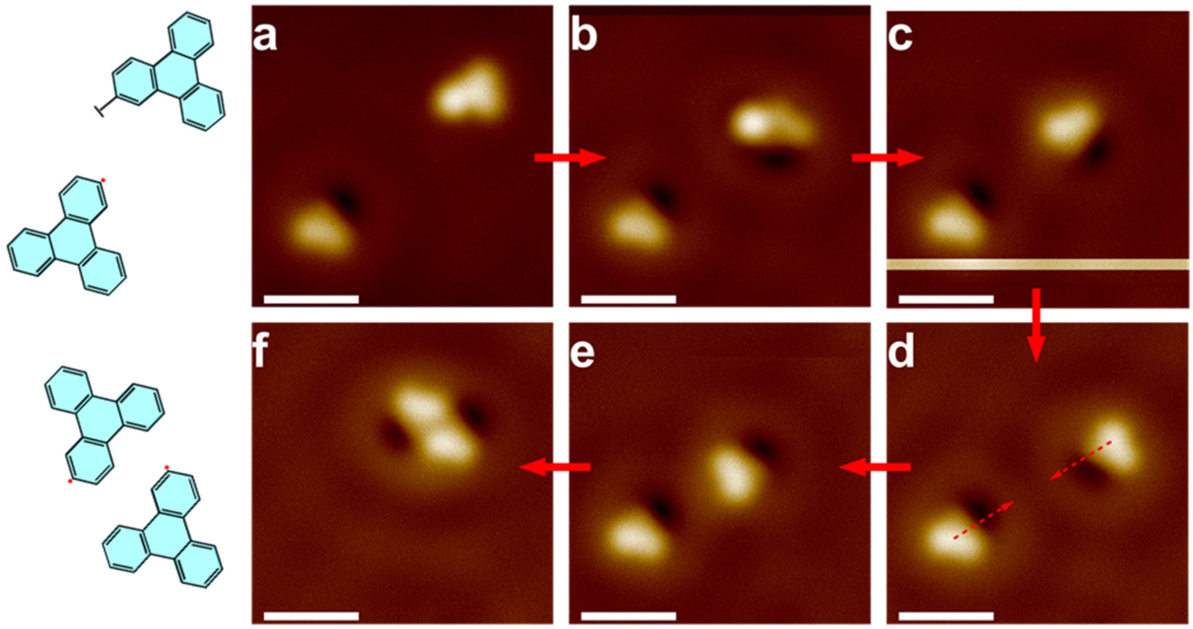

Supplementary Figure 9 | Force-driven manipulation of single T• radicals on NaCl(2ML)/Cu(111). a, STM image of an **IT** molecule (right) and a **T•** radical (left). Corresponding chemical structures are shown on the left. **b**, STM image after the deiodination of the **IT** molecule. **c**, STM image after removing the iodine atom next to the right triphenylene radical. **d**–**f**, STM images after moving the **T•** radicals to new positions with an I-modified tip as indicated with red dashed arrows in **d**. To move the molecules, the tip was first positioned at the edge of a molecule with the imaging parameters. Then, the tip–sample distance was reduced by increasing the tunneling current (typically 200 mV, 30–50 pA). Finally, the tip was moved along a predefined path across the molecule with an activated STM feedback, which often resulted in a displacement of the molecule in the direction of the tip movement. The two molecules formed a noncovalent dimer in **f** as depicted by the chemical structures on the left since the two radicals are not well aligned. The radicals were unintentionally picked up by the tip in the subsequent manipulation. Imaging parameters: 200 mV, 2 pA for all the STM images. Scale bar, 2 nm.

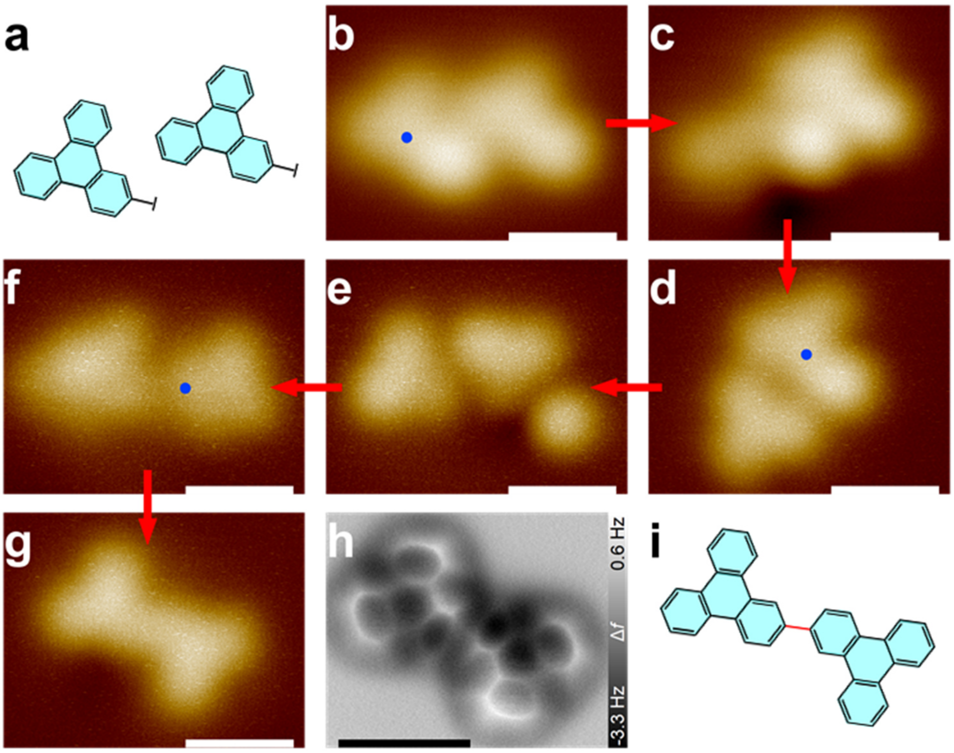

Supplementary Figure 10 | Detailed manipulation process for the fabrication of a triphenylene dimer *trans*-TT-1 on NaCl(2ML)/Cu(111). The blue dot in an STM image marks the position of the voltage pulses, and the result is shown in the next STM image. **a**,**b**, Chemical structure (**a**) and STM image (**b**) of a noncovalent **IT** dimer. **c**, STM image after the deiodination of the left **IT** molecule. The deiodination occurred at 1.88 V during a voltage ramp from 1.5 V to 2.0 V with a metal tip, ramp speed of 0.01 V/s, Δz = 300 pm relative to 200 mV, 2 pA. **d**, STM image after picking up the dissociated iodine atom by vertical manipulation. **e**, STM image after the deiodination of the right **IT** molecule. The deiodination occurred at 1.92 V during a voltage ramp from 1.5 V to 2.0 V with an I tip, ramp speed of 0.008 V/s, Δz = 300 pm relative to 500 mV, 1.5 pA. **f**, STM image after removing the second dissociated iodine atom by vertical manipulation. **g**–**i**, STM image (**g**), AFM image (**h**) and chemical structure (**i**) of the covalent triphenylene dimer ***trans*-TT-1** formed via the voltage pulse-induced (2.0 V, 100 ms, I tip) coupling between the two **T**$^{\bullet}$ radicals in **f**. Image **h** is a copy of Fig. 2d. Imaging parameters: (**b**,**c**) 200 mV, 2 pA. (**d**–**g**) 500 mV, 1.5 pA. Scale bar, 1 nm.

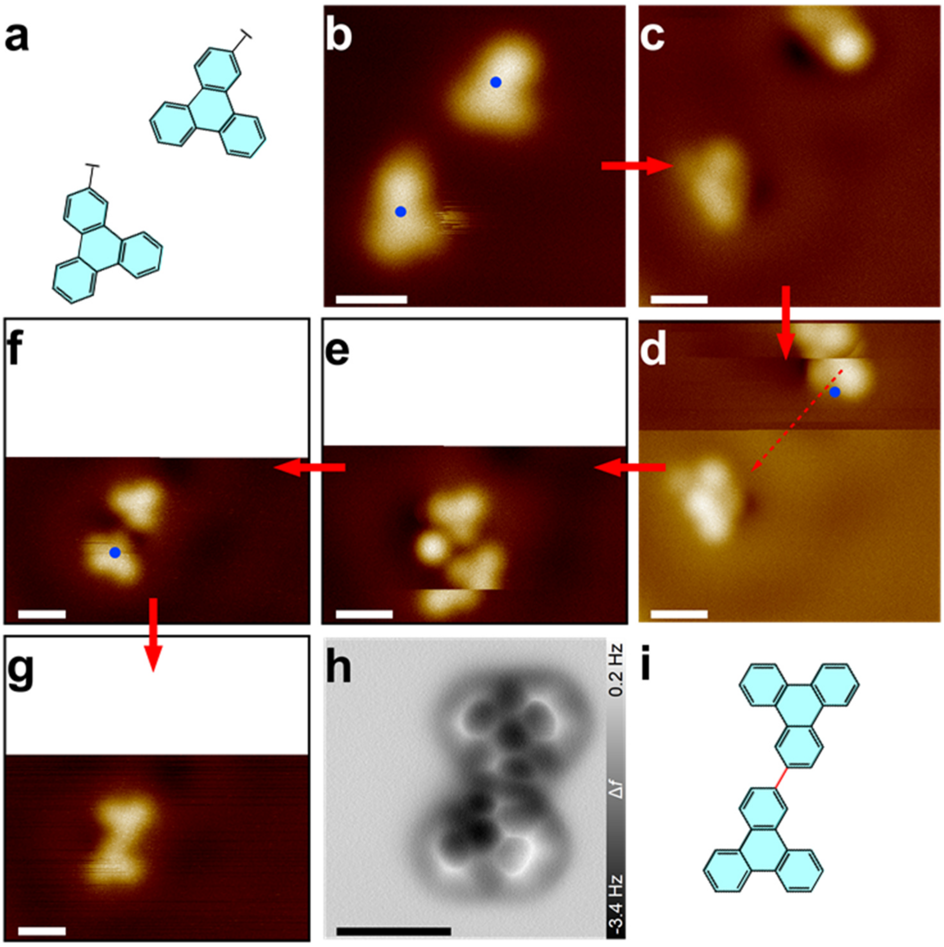

Supplementary Figure 11 | Detailed manipulation process for the fabrication of a *trans*-TT-2 dimer on NaCl(2ML)/Cu(111). The blue dots in an STM image mark the positions of the voltage pulses, and the result is shown in the next STM image. **a**,**b**, Chemical structure (**a**) and STM image (**b**) of two adjacent **IT** molecules. **c**, STM image after the deiodination of the two **IT** molecules. **d**, STM image after removing the dissociated iodine atom from the top molecule. **e**, STM image after moving the top **T•** radical to the other molecule with voltage pulses (2.0 V, 100 ms, I tip). **f**, STM image after removing the second dissociated iodine atom by vertical manipulation. **g**, STM image of the covalent ***trans*-TT-2** dimer formed by the voltage pulse-induced (2.0 V, 100 ms, I tip) coupling between the two **T•** radicals in **f**. **h**,**i**, Constant-height AFM image (**h**) and chemical structure (**i**) of ***trans*-TT-2**. Imaging parameters: (**b**–**e**) 200 mV, 2 pA. (**f**,**g**) 500 mV, 1.5 pA. (**h**) Tip height offset Δz = 70 pm relative to 500 mV, 1.5 pA. Scale bar, 1 nm.

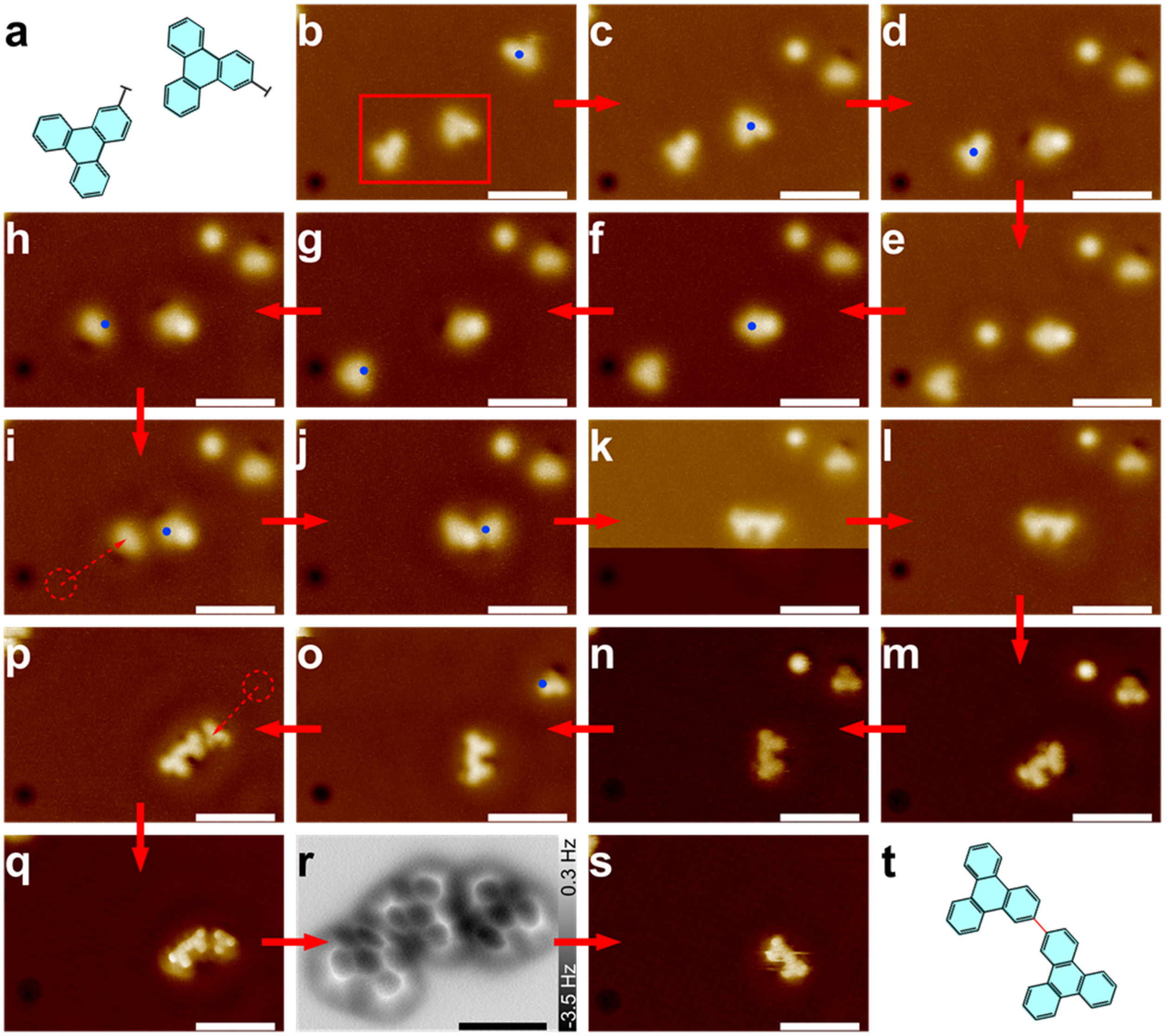

Supplementary Figure 12 | Detailed manipulation process for the fabrication of a triphenylene dimer *cis*-TT on NaCl(2ML)/Cu(111). The blue dot in an STM image marks the position of the voltage pulses, and the result is shown in the next STM image. **a**,**b**, Chemical structure (**a**) and STM image (**b**) of two adjacent **IT** molecules. A third **IT** molecule in the top-right corner acts as a marker. **c**–**e**, STM images after the deiodination of the right (**c**), the middle (**d**) and the left (**e**) **IT** molecules. **f**, STM image after removing the dissociated iodine atom from the left molecule. **g**, The middle $\mathbf{T}^\bullet$ radical switched from free state to bound state by a voltage pulse (2.0 V, 20 ms). **h**,**i**, The left $\mathbf{T}^\bullet$ radical was moved to the middle one by voltage pulses (2.0 V, 20 ms, metal tip). The displacement of the left $\mathbf{T}^\bullet$ radical with respect to the initial position (red dashed circle) is indicated with a red dashed arrow in **i**. **j**, Repositioning and reorientation of the two middle $\mathbf{T}^\bullet$ radicals by voltage pulses (2.0 V, 20 ms, metal tip). **k**, A covalent ***cis*-TT** dimer was formed by a voltage pulse of 2.0 V, 20 ms with a metal tip. Meanwhile, the middle iodine atom jumped to the STM tip causing a tip change. **l**, STM image with an I-modified tip. **m**,**n**, The dimer was rotated unintentionally during imaging with a CO tip. **o**, The iodine atom in the top-right corner was picked up by the tip. **p**, The right $\mathbf{T}^\bullet$ radical was moved to the dimer by voltage pulses (2.0 V, 20 ms) with the I tip. **q**, STM image of the stabilized dimer with a CO tip. **r**, Constant-height AFM image of the stabilized dimer. **s**, STM image of the dimer after removing the right molecule. **t**, Chemical structure of the ***cis*-TT** dimer. Imaging parameters: (**b**–**l**,**o**,**s**) 500 mV, 1.5 pA. (**m**) 600 mV, 1.5 pA. (**n**) 600 mV, 1.3 pA. (**p**) 200 mV, 1.5 pA. (**q**) 200 mV, 2 pA. (**r**) Tip height offset Δz = 70 pm relative to 500 mV, 1.5 pA. Scale bars, 3 nm (**b**-**q** and **s**) and 1 nm (**r**).

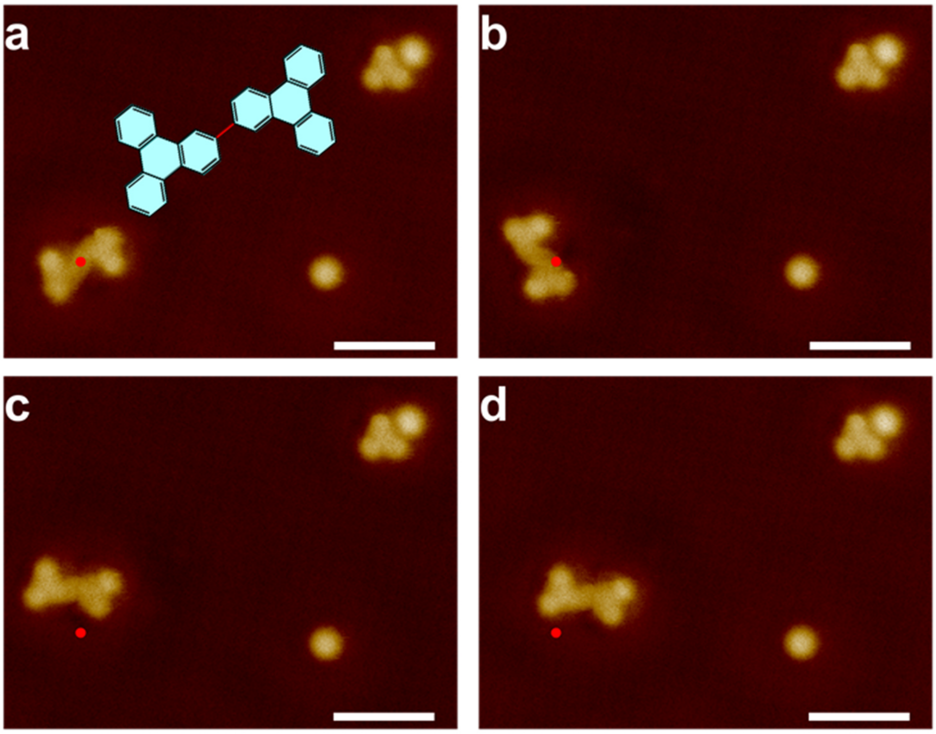

Supplementary Figure 13 | Voltage pulse-induced manipulation of a covalent triphenylene dimer *trans*-TT-1 on NaCl(2ML)/Cu(111). a, STM image of a covalent dimer ***trans*-TT-1** (left, cf. the inset chemical structure), an iodine-anchored **T•** radical (top-right) and an iodine atom (bottom-right). **(b–d)** STM images showing that the ***trans*-TT-1** dimer was moved away from its original position (marked with a red dot) by voltage pulses (2.0 V, 30 ms, CO tip). Imaging parameters: 500 mV, 2 pA for all the STM images. Scale bar, 2 nm.

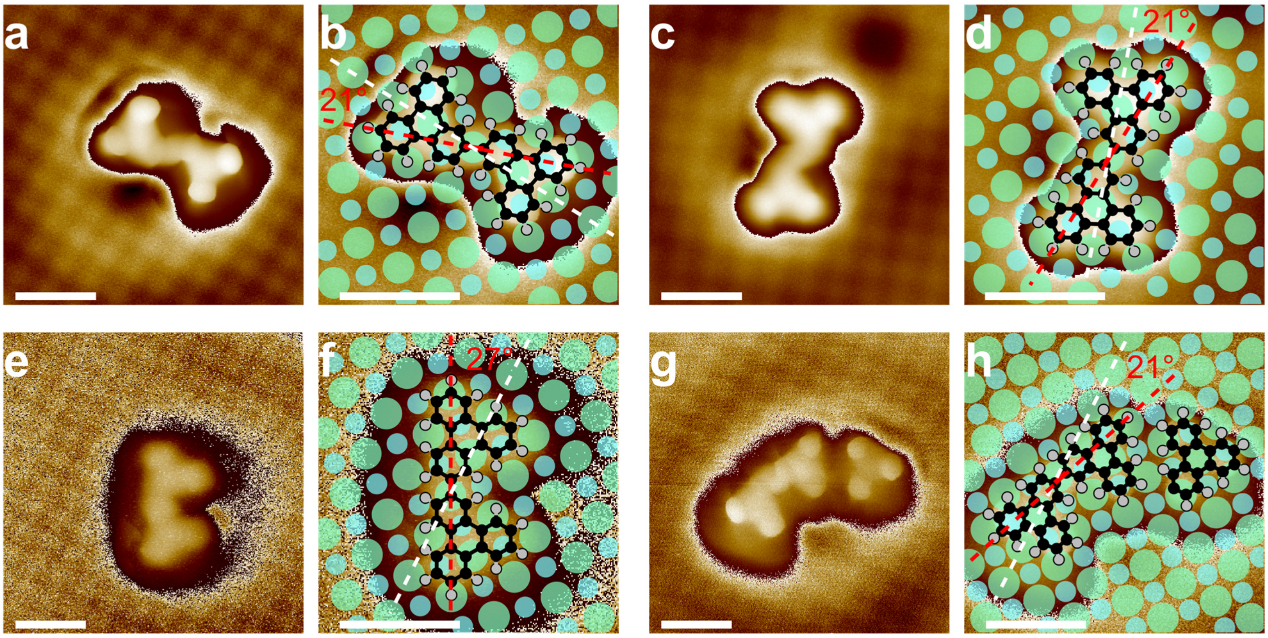

Supplementary Figure 14 | Adsorption positions of covalent TT dimers on NaCl(2ML)/Cu(111). a, Atomically resolved STM image of a ***trans*-TT-1** dimer. The image contrast was adjusted separately for the molecule and the surface. **b**, The same image as **a** on which a NaCl lattice and a molecular model are superimposed. **c**–**h**, High-resolution STM images and fitting of molecular models for ***trans*-TT-2** (**c**,**d**) and ***cis*-TT** dimers (**e**–**h**). Elements are color-coded: Cl (light green), Na (light blue), C (black), H (grey). Imaging parameters: (**a**,**b**,**g**,**h**) 200 mV, 2 pA. (**c**,**d**) 500 mV, 2 pA. (**e**,**f**) 600 mV, 1.3 pA. Scale bar, 1 nm.

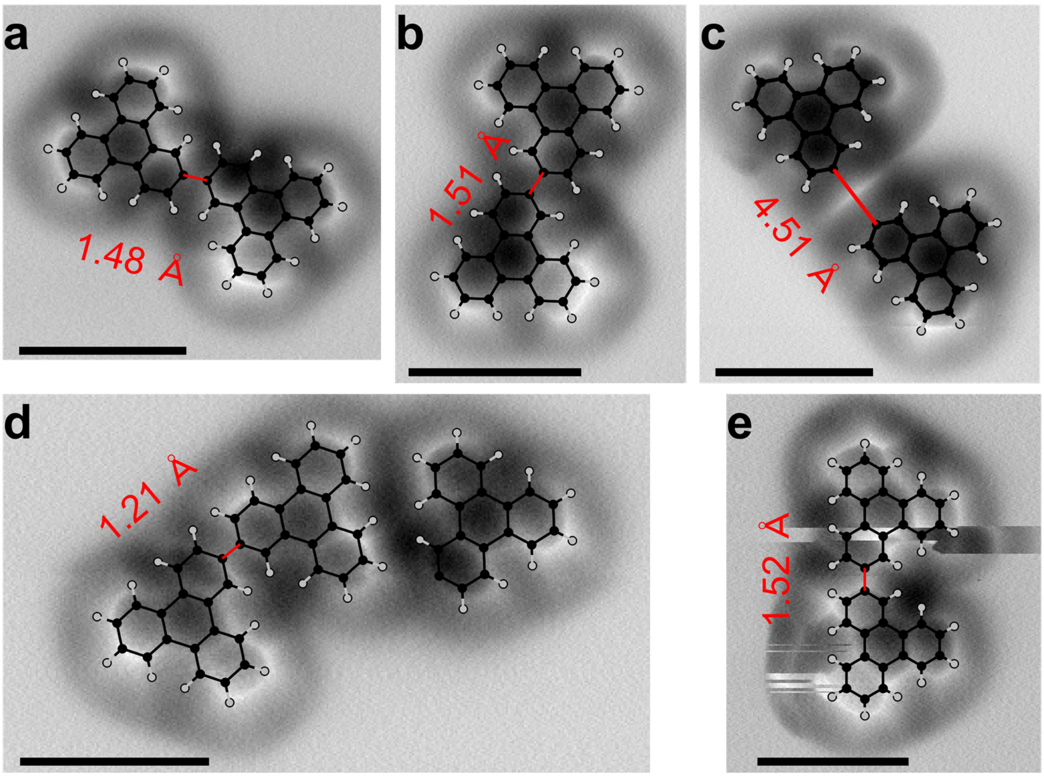

Supplementary Figure 15 | Bond-length measurement of the newly formed C–C bonds in the TT dimers and the C–I–C bond in the TIT structure. a, Two triphenylene radical models were fitted onto the constant-height AFM image of a ***trans*-TT-1** dimer. The bond length of the newly formed C–C bond (red) was determined by the distance between the two carbon atoms of the connection. **b**–**e**, Same method was exploited to acquire the bond length of the new C–C bonds in ***trans*-TT-2** (**b**) and ***cis*-TT** (**d**,**e**) dimers and the total length of the C–I–C bond in a **TIT** structure (**c**). The adsorption of the ***cis*-TT** dimer on NaCl is unstable and it was often rotated by the tip during imaging as shown in **e**. The ***cis*-TT** dimer was stabilized by another triphenylene radical in **d**. AFM images **a**–**d** are copies of Supplementary Figs. 10h and 11h, Fig. 2f and Supplementary Fig. 12r, respectively. (**e**) Tip height offset Δz = 80 pm relative to 500 mV, 1.5 pA. Scale bar, 1 nm.

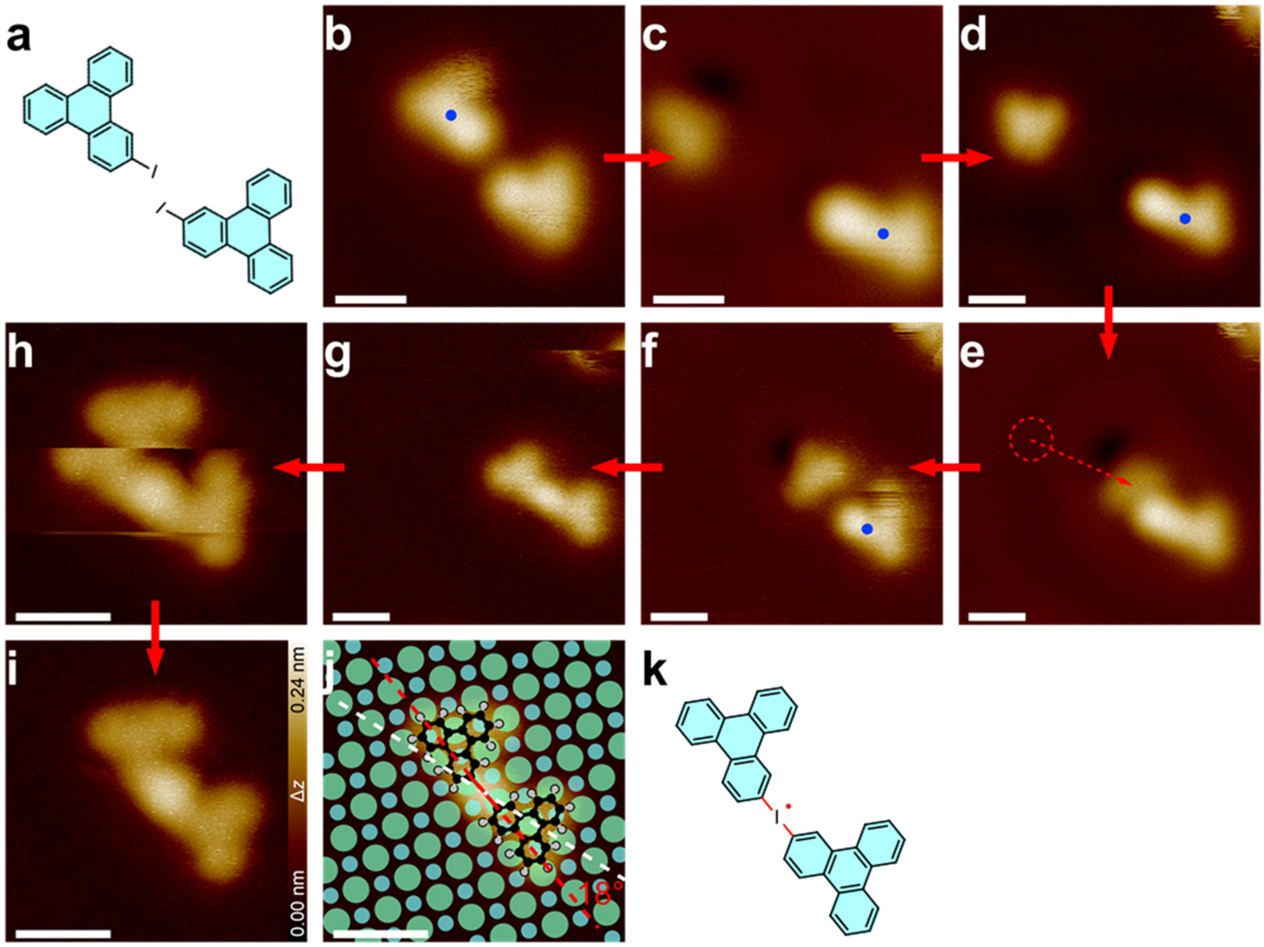

Supplementary Figure 16 | Detailed manipulation process for the fabrication of a TIT structure on NaCl(2ML)/Cu(111). The blue dot in an STM image marks the position of the voltage pulse, and the result is shown in the next STM image. **a**,**b**, Chemical structure (**a**) and STM image (**b**) of two adjacent **IT** molecule. **c**, STM image after the deiodination of the left **IT** molecule. The dissociated iodine atom attached to the right **IT** molecule. The deiodination occurred at about 2.0 V during a voltage ramp from 1.5 V to 2.0 V with a metal tip, ramp speed of 0.01 V/s, Δz = 300 pm relative to 200 mV, 2 pA. **d**, STM image showing that the left **T•** radical switched from bound state to free state by a voltage ramp (1.5–2.0 V, metal tip) over the right molecule. **e**, STM image after the **T•** radical shifted to the right molecule induced by a voltage ramp (1.5–2.0 V, metal tip) over the right molecule. The displacement of the **T•** radical with respect to the previous position (red dashed circle) is indicated with a red dashed arrow. **f**, STM image after picking up the dissociated iodine atom between the two molecules by vertical manipulation. **g**, STM image of the sandwich structure **TIT** formed by the coupling between the triphenylene radical and the **IT** molecule triggered by a voltage ramp (1.5–2.0 V, I tip). **h**,**i**, STM images of the **TIT** structure with a CO tip. The **TIT** structure was accidently rotated by the tip during imaging. **j**, The same image as **i** on which a NaCl lattice and molecular models are superimposed. **k**, Chemical structure of **TIT**. Imaging parameters: (**b**–**e**) 200 mV, 2 pA. (**f**,**g**) 500 mV, 1.5 pA. (**h**,**i**,**j**) 600 mV, 1.3 pA. Scale bar, 1 nm.

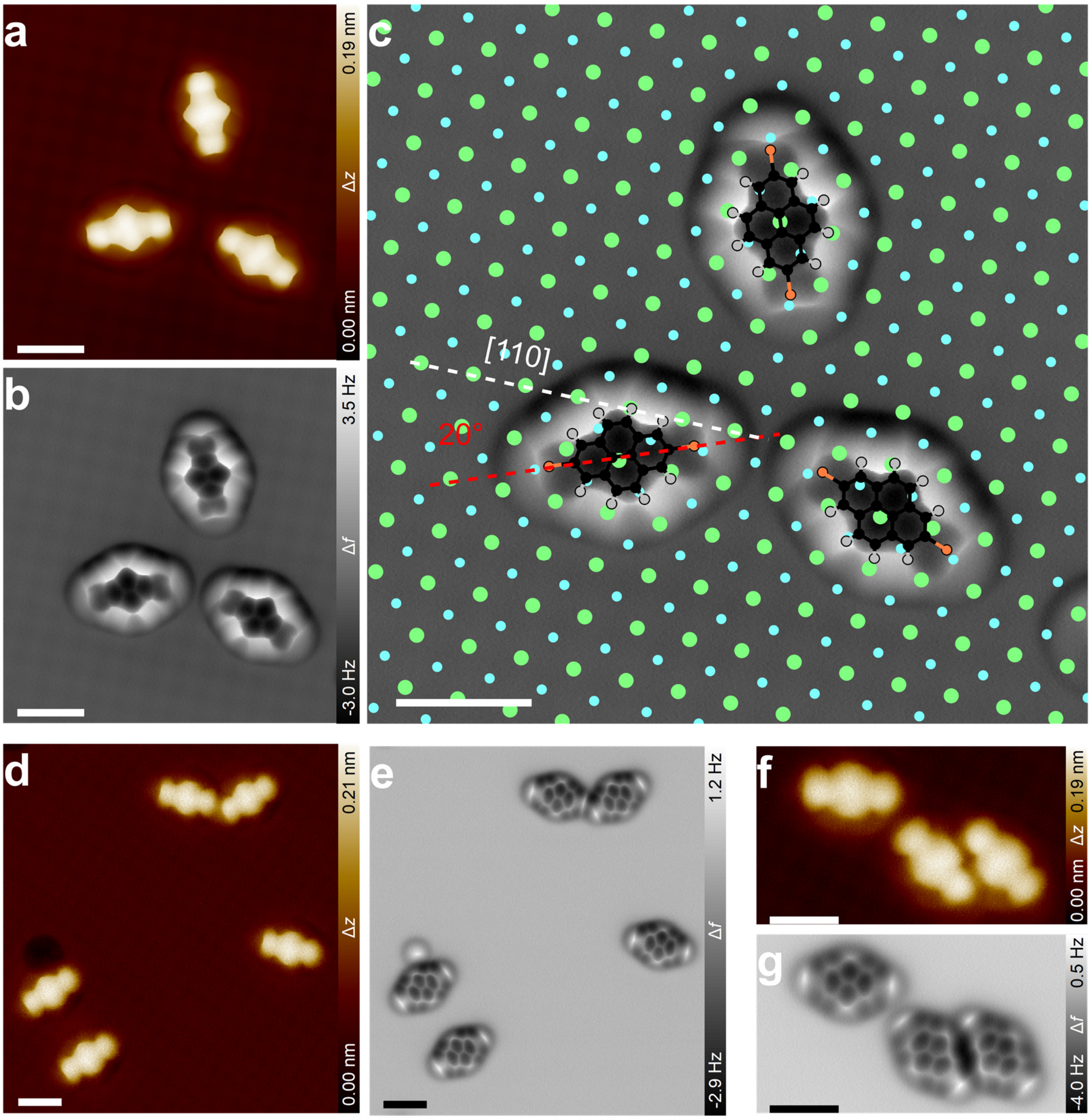

Supplementary Figure 17 | High-resolution imaging of DBP molecules on NaCl(2ML)/Cu(111). a,**b**, Bond-resolved STM (**a**) and AFM (**b**) images of three **DBP** molecules simultaneously recorded in constant-current mode. **c**, Same image as **b** on which a NaCl lattice and three molecular models were superimposed. Elements are color-coded: Cl (light green), Na (light blue), C (black), H (grey), Br (orange). **d**–**g**, Constant-current STM images (**d**,**f**) and constant-height AFM images (**e**,**g**) of a few more **DBP** molecules on NaCl(2ML)/Cu(111). Imaging parameters: (**a**–**c**) 200 mV, 2 pA. (**d**,**f**) 500 mV, 2 pA. (**e**,**g**) Tip height offset Δ*z* = 100 pm relative to 500 mV, 2 pA. Scale bar, 1 nm.

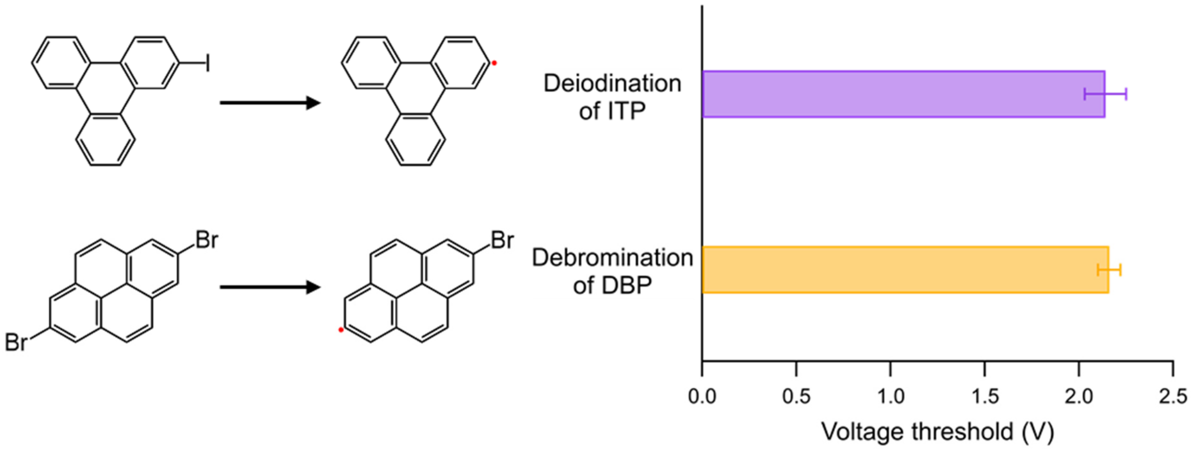

Supplementary Figure 18 | Voltage thresholds for the dehalogenation of IT and DBP on NaCl(2ML)/Cu(111). A copper tip was exploited to trigger the reactions. The tip was lifted up by 300 pm relative to 500 mV, 2 pA at the center of a molecule. An $I(V)$ spectrum was subsequently recorded (parameters: 1.5–2.3 V, ramp speed of 0.016 V/s, Δz = 300 pm relative to 500 mV, 2 pA, metal tip). A sudden change in current often indicates the dissociation of halogens, which was confirmed by STM imaging. In total, ten **DBP** molecules and seven **IT** molecules were counted. The average threshold voltages are very similar for the deiodination of **IT** (2.14 ± 0.11 V) and the debromination of **DBP** (2.16 ± 0.06 V) on NaCl(2ML)/Cu(111). The error bars represent the standard deviation.

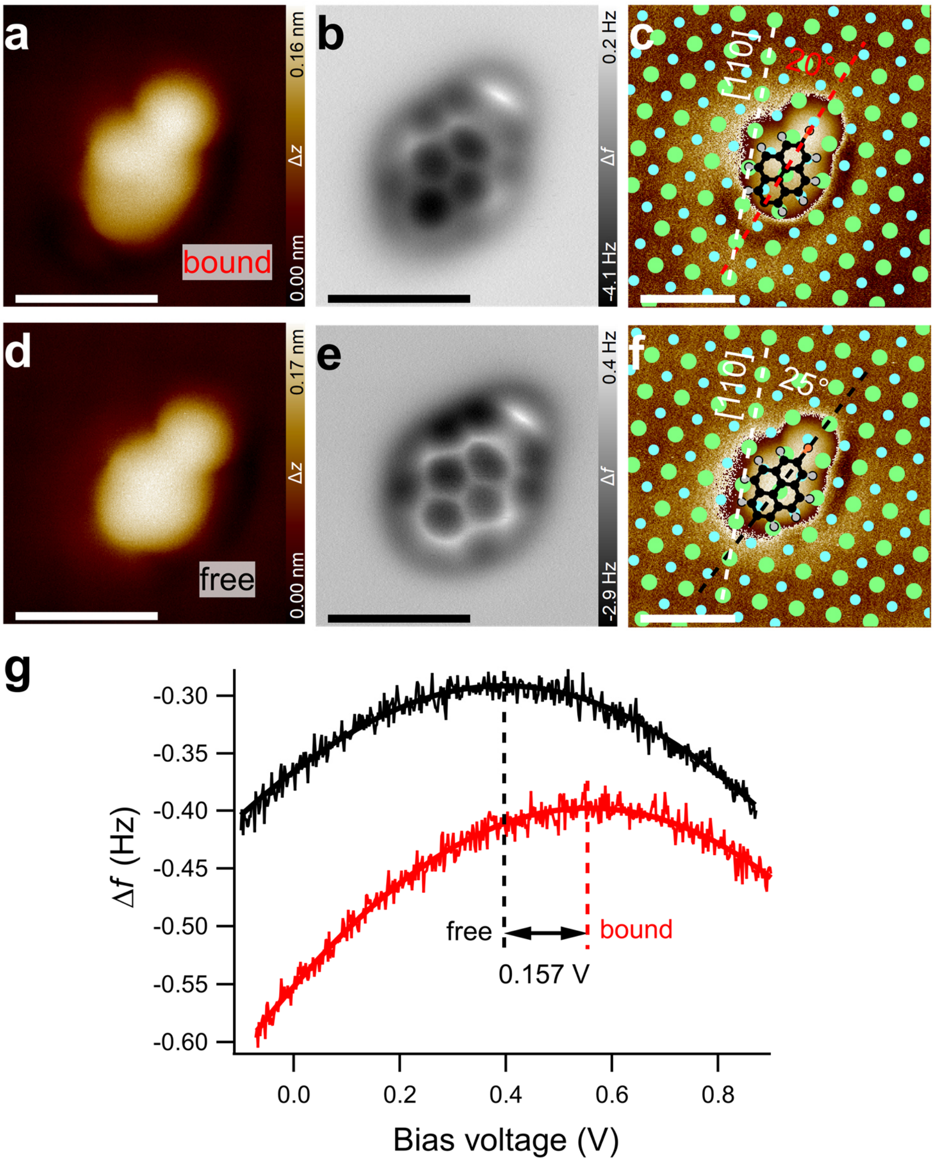

Supplementary Figure 19 | High-resolution imaging and Kelvin probe force spectroscopy (KPFS) of a single bromopyrene monoradical in bound and free states on NaCl(2ML)/Cu(111). a,**b**, STM image (**a**) and (**b**) constant-height AFM image of a bromopyrene monoradical in bound state. **c**, Atomically resolved STM image for the bound state superimposed with a NaCl lattice and a molecular model. **d**,**e**, STM image (**d**) and constant-height AFM image (**e**) of the bromopyrene monoradical in free state induced by a short (< 500 ms) voltage pulse of −0.5 V. **f**, Atomically resolved STM image for the free state superimposed with a NaCl lattice and a molecular model. **g**, KPFS spectra obtained over the radical site for the bound (red) and free (black) states. Image contrast in **c**,**f** is separately adjusted for the surface and the molecule. Imaging parameters: (**a**,**c**,**d**,**f**) 500 mV, 2 pA. (**b**,**e**) Tip height offset Δz = 80 pm relative to 500 mV, 2 pA. Scale bar, 1 nm.

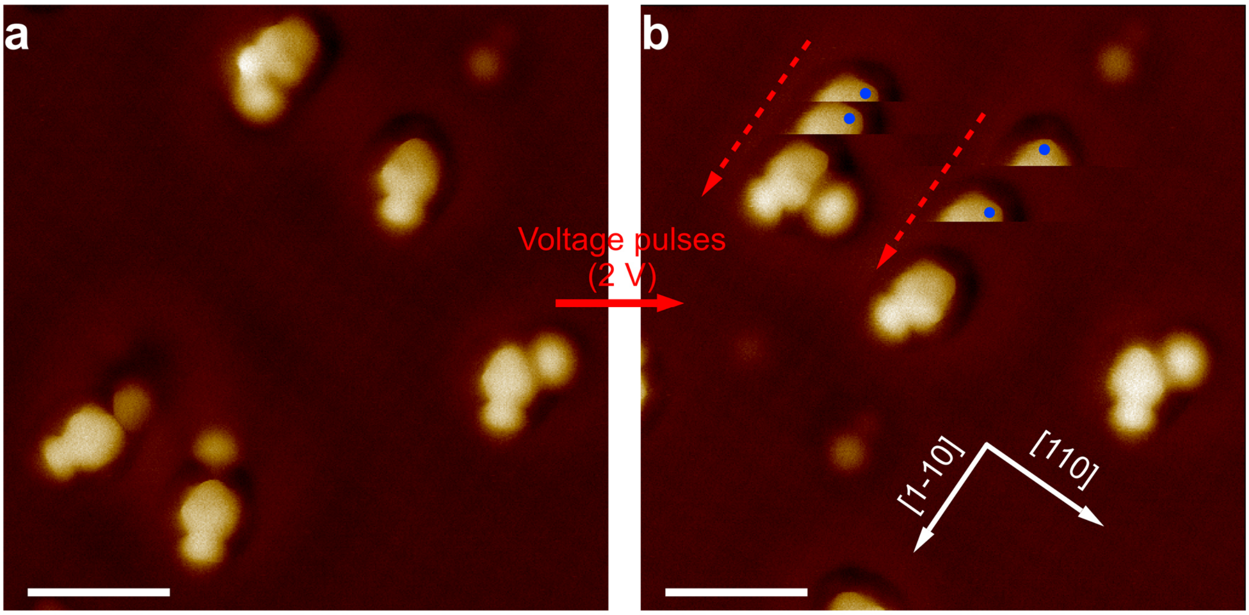

Supplementary Figure 20 | Voltage-pulse-induced manipulation of 2-bromopyrene monoradicals on NaCl(2ML)/Cu(111). a, STM image of five partially debrominated bromopyrene monoradicals. **b**, STM image showing the directional manipulation (red dashed arrows) of the top two molecules by applying voltage pulses (2.0 V, 10 ms, CO tip) over the radical sites (blue dots). Imaging parameters: (**a**,**b**) 500 mV, 2 pA. Scale bar, 2 nm.

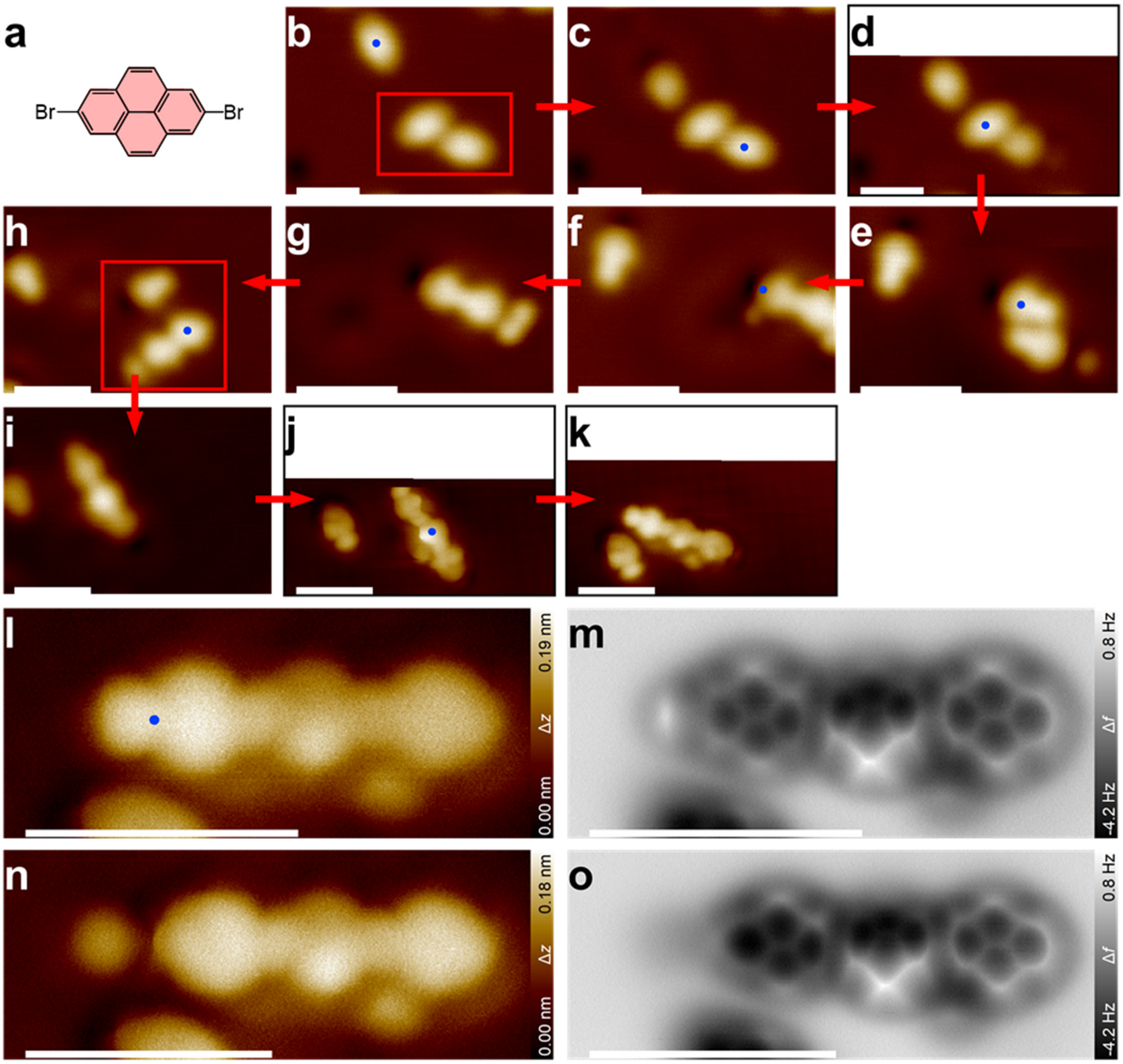

Supplementary Figure 21 | Detailed manipulation process for the fabrication of a PPP$^{2•}$ trimer on NaCl(2ML)/Cu(111). The blue dot in an STM image marks the position of the voltage pulses, and the result is shown in the next STM image. **a**, Chemical structure of **DBP**. **b**, STM image of three **DBP** molecules. **c**–**e**, STM images after the partial debromination of the left (**c**), right (**d**) and middle (**e**) **DBP** molecules by voltage pulses of about 2.2 V. **f**, A covalent pyrene dimer **PP$^{2•}$** was formed via the voltage pulse-induced (3.0 V, 10 ms, Δz = 300 pm, Br tip) coupling between the right two molecules. **g**, The **PP$^{2•}$** dimer was moved by a voltage pulse (3.0 V, 10 ms, Δz = 300 pm, Br tip). **h**, The **PP$^{2•}$** dimer was unintentionally moved to a new place by the tip when trying to pick up the bromine atoms next to it. **i**, A covalent pyrene trimer was formed via the coupling between the **PP$^{2•}$** dimer and an adjacent bromopyrene monoradical triggered by a voltage pulse (3.0 V, 10 ms, Δz = 300 pm, metal tip). **j**, High-resolution STM image of the trimer with a CO tip. **k**, The pyrene trimer was laterally manipulated by a voltage pulse (3.0 V, 10 ms, Δz = 300 pm, CO tip). **l**,**m**, STM (**l**) and constant-height AFM (**m**) images of the pyrene trimer. A C–Br bond remains at the left end. **n**,**o**, STM (**n**) and constant-height AFM (**o**) images of the **PPP$^{2•}$** trimer formed by cleaving the C–Br bond (2.3 V) of the structure in **l**. Image **o** is a copy of Fig. 3e. Imaging parameters: (**b**–**d**) 200 mV, 2 pA. (**e**–**l** and **n**) 500 mV, 2 pA. (**m**) Δz = 100 pm relative to 500 mV, 2 pA. Scale bar, 2 nm.

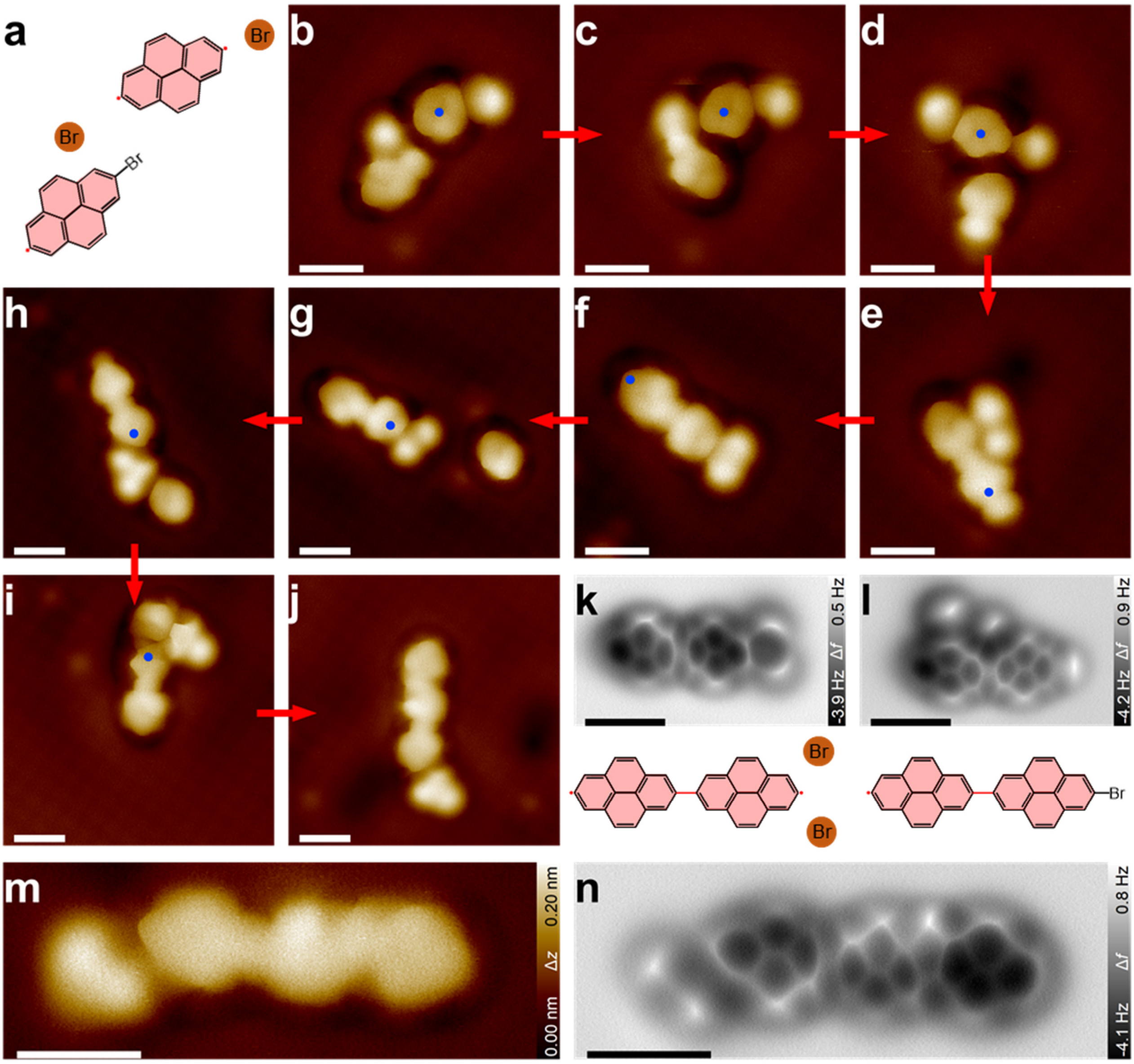

Supplementary Figure 22 | Detailed manipulation process for the fabrication of a d-PPP$^{3•}$ trimer on NaCl(2ML)/Cu(111). The blue dot in an STM image marks the position of the voltage pulses, and the result is shown in the next STM image. **a**,**b**, Chemical structure (**a**) and STM image (**b**) of a pyrene diradical and a bromopyrene monoradical. **c**,**d**, STM images showing the lateral manipulation of the two molecules by applying voltage pulses (3.0 V, 10 ms, Δz = 300 pm, relative to 500 mV, 2 pA, CO tip) over the diradical. **e**, A partially debrominated pyrene dimer was formed by a voltage pulse (3.0 V, 10 ms, Δz = 300 pm, CO tip). **f**, The C–Br bond at the bottom end was cleaved and two Br atoms were left in the vicinity after a few pulses (3.0 V, 10 ms, Δz = 300 pm, CO tip). The third Br atom was out of the frame. **g**, The **PP$^{2•}$** dimer was moved to another pyrene diradical **P$^{2•}$** by voltage pulses (2.0 V, 10 ms, CO tip). **h**,**i**, The **PP$^{2•}$** dimer and the pyrene diradical were relocated by a few voltage pulses (3.0–3.5 V, 10 ms, Δz = 300 pm, CO tip). **j**, STM image of the covalent trimer formed by a few voltage pulses (3.5 V, 10 ms, Δz = 300 pm, CO tip). **k**,**l**, Constant-height AFM images and chemical structures corresponding to **f** and **e**, respectively. **m**,**n**, STM (**m**) and constant-height AFM (**n**) images of the defective trimer **d-PPP$^{3•}$** in **j**. One of the three Br atoms on the left side was removed beforehand by vertical manipulation. Imaging parameters: (**b**–**j** and **m**) 500 mV, 2 pA. (**k**,**l**,**n**) Tip height offset Δz = 100 pm relative to 500 mV, 2 pA. Scale bar, 1 nm.

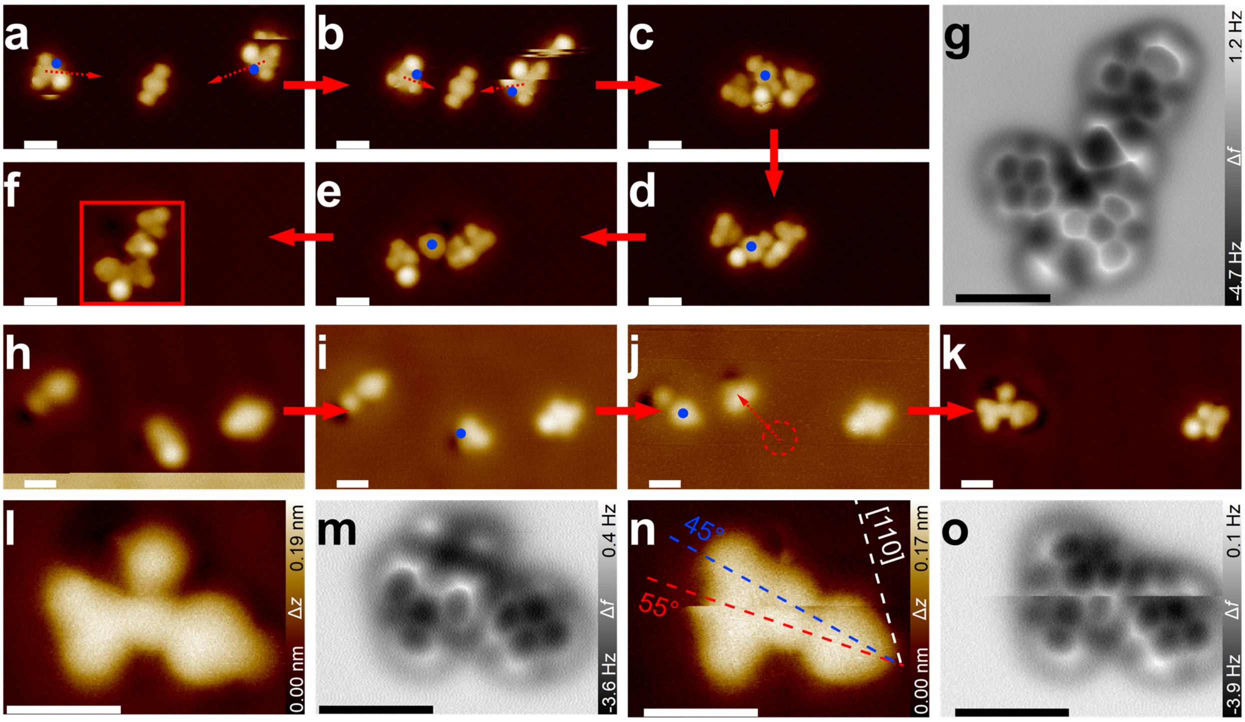

Supplementary Figure 23 | Detailed manipulation process for the fabrication of a covalent triphenylene-pyrene (TP•-1) dimer on NaCl(2ML)/Cu(111). The blue dots in an STM image mark the positions of the voltage pulses, and the result is shown in the next STM image. **a**, STM image of two **IT** molecules (left and right) and a **DBP** molecule (middle). **b**,**c**, The two **IT** molecules were moved to the **DBP** molecule by voltage pulses (1.5 V, 10 ms, CO tip). The displacement of the **IT** molecules is indicated in **a** and **b** with red dashed arrows. The left **IT** molecule underwent deiodination during the manipulation. **d**, STM image after cleaving the left C–Br bond of the **DBP** molecule using a voltage pulse (2.0 V, 10 ms, CO tip). **e**, The second C–Br bond of the **DBP** molecule was cleaved by another voltage pulse (2.5 V, 10 ms, CO tip). **f**, A covalent **TP•-1** dimer was formed by a few voltage pulses (2.0 V, 10 ms, CO tip). **g**, Constant-height AFM image of the structures in the red rectangle in **f**. **h**, STM image of a partially debrominated **DBP** molecule (left) and two deiodinated **IT** molecules (middle and right). **i**, STM image after removing the dissociated iodine atom from the middle molecule by vertical manipulation. **j**, The middle **T•** radical was moved to the left bromopyrene monoradical by voltage pulses (2.0 V, 10 ms, I tip). The displacement of the **T•** radical with respect to the previous position (red dashed circle) is indicated with a red dashed arrow. **k**, Another **TP•-1** dimer was formed by voltage pulses (3.0 V, 10 ms, Δz = 300 pm, I tip). **l**,**m**, STM (**l**) and AFM (**m**) images of the **TP•-1** dimer. **n**,**o**, STM (**n**) and AFM (**o**) images of the **TP•-1** dimer after removing the CO molecule and the Br atom attached to the dimer. Imaging parameters: (**a**–**f**) and (**h**,**i**,**k**,**l**,**n**) 500 mV, 2 pA. (**j**) 500 mV, 1.5 pA. (**g**,**m**,**o**) Tip height offset Δz = 100 pm relative to 500 mV, 2 pA. Scale bar, 1 nm.

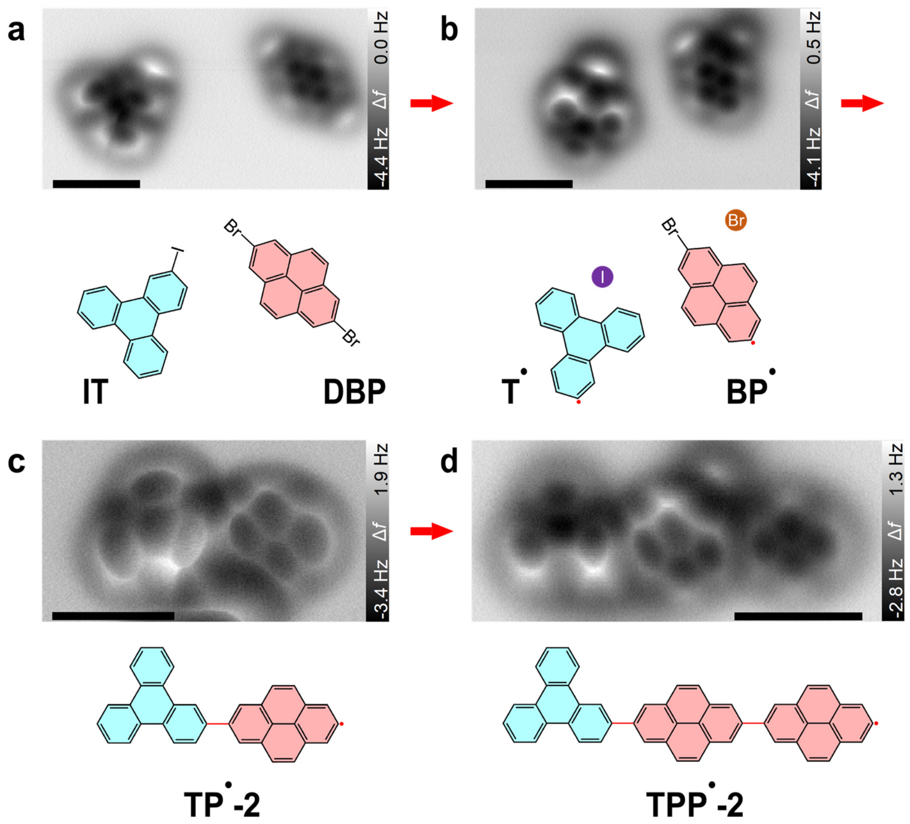

Supplementary Figure 24 | Tip-induced fabrication of a triphenylene-pyrene dimer TP•-2 and a triphenylene-bipyrene trimer TPP•-2 on NaCl(2ML)/Cu(111). A few representative AFM images illustrate the essential steps for the manipulation. **a**, AFM image of an **IT** and a **DBP** molecule. **b**, AFM image after deiodination of the **IT** molecule and partial debromination of the **DBP** molecule by voltage pulses (2.0 V, 10 ms, CO tip). **c**, A **TP•-2** dimer resulted from the cross-coupling between a triphenylene radical and a pyrene radical triggered by a voltage pulse (3.0 V, 10 ms, Δ*z* = 300 pm, CO tip). **d**, A **TPP•-2** trimer resulted from the coupling between a **TP•-2** dimer and a pyrene radical triggered by voltage pulses (3.0 V, 10 ms, Δ*z* = 300 pm, I tip). The images are collected from different series. Image **d** is a copy of Fig. 4e. Tip height offset Δ*z* = 100 pm (**a**,**c**) and 90 pm (**b**) relative to 500 mV, 2 pA. Scale bar, 1 nm.

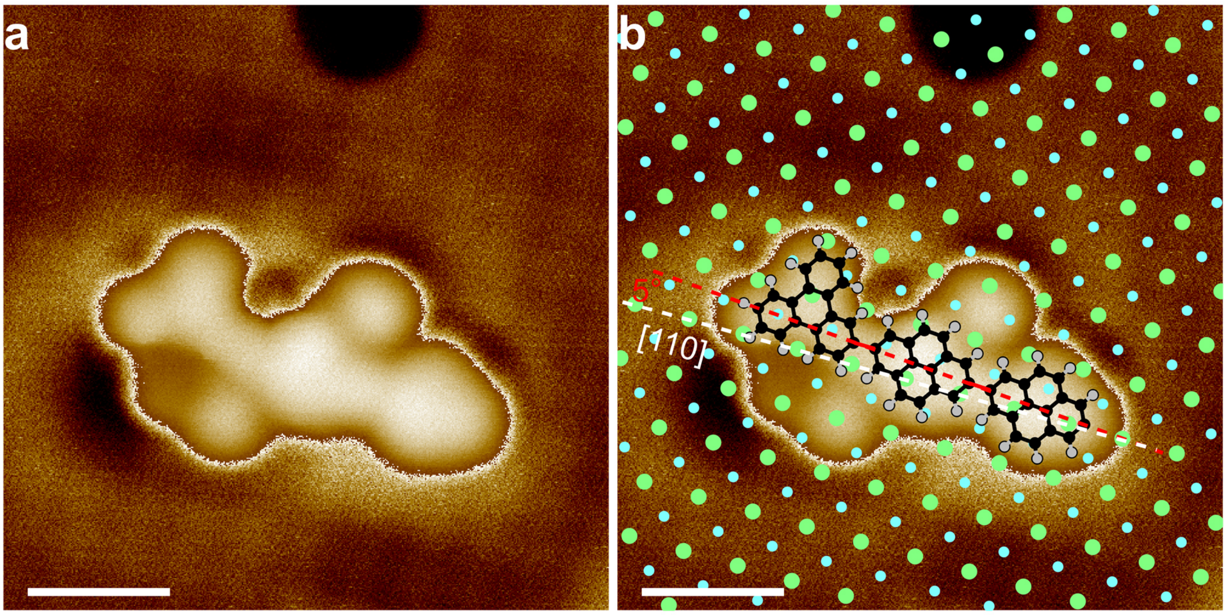

Supplementary Figure 25 | Adsorption position of a TPP$^{\bullet}$-2 trimer on NaCl(2ML)/Cu(111). a, STM image of a **TPP$^{\bullet}$-2** trimer with atomic resolution of the surrounding surface. **b**, The same STM image as **a** on which a NaCl lattice and molecular models are superimposed. Elements are color-coded: Cl (light green), Na (light blue), C (black), H (grey). Imaging parameters: 500 mV, 2 pA. Scale bar, 1 nm.

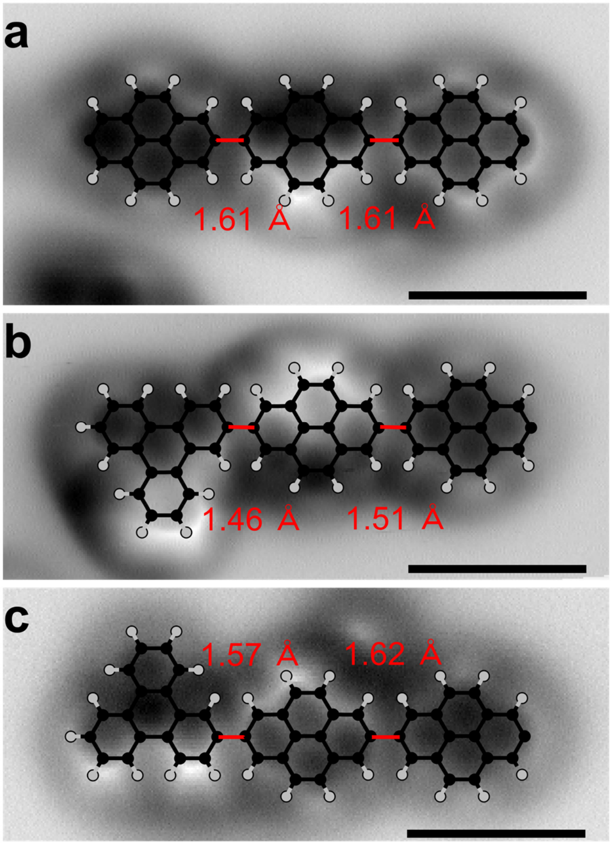

Supplementary Figure 26 | Bond-length measurements of the newly formed C–C bonds in the PPP$^{2•}$ and TPP$^{•}$ trimers. Molecular models of pyrene diradical and triphenylene radical were first fitted onto the constant-height AFM images. The bond length of the new C–C bonds (red) was determined by the distance between the two carbon atoms connected by a red bond. AFM images **a**–**c** are copies of Figs. 3e, 4d and 4e, respectively. Scale bar, 1 nm.

Supplementary references

1. Steurer, W., Fatayer, S., Gross, L. & Meyer, G. Probe-based measurement of lateral single-electron transfer between individual molecules. *Nat. Commun.* **6,** 8353 (2015).
2. Gross, L. *et al.* Bond-order discrimination by atomic force microscopy. *Science* **337,** 1326-1329 (2012).
3. Weymouth, A. J., Hofmann, T. & Giessibl, F. J. Quantifying molecular stiffness and interaction with lateral force microscopy. *Science* **343,** 1120-1122 (2014).
4. Mönig, H. Copper-oxide tip functionalization for submolecular atomic force microscopy. *Chem. Commun.* **54,** 9874-9888 (2018).
